# Mechanical properties of white matter tracts in aging assessed via anisotropic MR elastography

**DOI:** 10.1162/IMAG.a.1156

**Published:** 2026-03-05

**Authors:** Diego A. Caban-Rivera, L. Tyler Williams, Matthew D.J. McGarry, Daniel R. Smith, Elijah E.W. Van Houten, Keith D. Paulsen, Philip V. Bayly, Curtis L. Johnson

**Affiliations:** Department of Biomedical Engineering, University of Delaware, Newark, DE, United States; Thayer School of Engineering, Dartmouth College, Hanover, NH, United States; Emory Sports Performance And Research Center (SPARC), Flowery Branch, GA, United States; Emory Sports Medicine Center, Atlanta, GA, United States; Department of Orthopaedics, Emory University School of Medicine, Atlanta, GA, United States; Département de Génie Mécanique, Universite de Sherbrooke, Sherbrooke, QC, Canada; Dartmouth-Hitchcock Medical Center, Lebanon, NH, United States; Mechanical Engineering and Materials Science, Washington University in St. Louis, St. Louis, MO, United States

**Keywords:** elastography, stiffness, anisotropy, aging, white matter, brain

## Abstract

Magnetic resonance elastography (MRE) is a promising neuroimaging technique to probe tissue microstructure through mechanical properties, such as stiffness, and which has revealed widespread softening in the aging brain and in neurological disorders. Traditional MRE approaches assume mechanical isotropy. However, white matter is known to be anisotropic from aligned, myelinated axonal bundles, which can lead to uncertainty in mechanical property estimates in these areas from isotropic MRE. Recent advances in anisotropic MRE now allow for estimation of shear and tensile anisotropy, along with substrate shear modulus, in white matter tracts. The objective of this study was to investigate age-related differences in anisotropic mechanical properties in human brain white matter for the first time. Anisotropic mechanical properties in major white matter tracts were found to be significantly lower in older adults (mean 68.5 ± 5.5 years; range 57–82) compared to young adults (mean 25.4 ± 2.2 years; range 22–30), with average property differences ranging between 0.028–0.111 for shear anisotropy and between 0.140–0.350 for tensile anisotropy. Stiffness perpendicular to the axonal fiber direction was significantly lower in older adults in the anterior thalamic radiation (2.78 ± 0.21 vs. 3.00 ± 0.15 kPa; p < 0.001) and forceps minor (2.61 ± 0.23 vs. 2.94 ± 0.23 kPa; p < 0.001) fibers, while stiffness parallel to fiber direction was lower in most tracts with an average difference between groups of 0.36 kPa or 12.1%, reflected by the widespread lower shear anisotropy observed in older adults. Comparing anisotropic MRE metrics with multiple measures of white matter microstructure from diffusion tensor imaging using tract-based spatial statistics, we observed anisotropic MRE measures further differentiated young and older adults in a logistic regression analysis, and widespread differences between mechanical anisotropy and diffusion tensor imaging (DTI) parameters were observed in the voxel-wise analysis. These results suggest that aging effects seen in anisotropic MRE measures are not overly redundant with aging effects on diffusion measures, and the addition of anisotropic MRE measures could further describe differences between the white matter of young and older adult populations. Anisotropic MRE provides a new tool for studying white matter microstructure with mechanical properties in aging and neurodegeneration.

## 1 Introduction

Magnetic resonance elastography (MRE) is a noninvasive MR imaging technique that produces quantitative maps of viscoelastic mechanical properties in soft biological tissues (Hiscox et al., 2016; Muthupillai et al., 1995; Sack, 2022). Brain MRE experiments are performed with an external vibration source used to propagate shear waves through the skull into the brain, inducing micron-level displacements. The MRI-captured wave motion images are then inverted to generate maps of mechanical properties of the tissue such as mechanical stiffness and viscosity (Manduca et al., 2021), providing insights into the changes in the structural health of brain tissue. In the human brain, MRE has been utilized in several studies revealing softer neural tissue in older age and in neurological diseases, including Alzheimer’s disease and other dementias (Delgorio et al., 2023; Hiscox et al., 2020; Huston et al., 2016; Murphy et al., 2019; Pavuluri et al., 2023). Through preclinical animal models, changes to mechanical properties of brain tissue have been shown to relate to the composition and organization of microscale elements, indicated by significant correlations with neuronal density and myelin content (Freimann et al., 2013; Schregel et al., 2012), while studies have highlighted the mechanical importance of myelination (Weickenmeier et al., 2016, 2017). MRE measures of stiffness and viscosity can assess these microscale changes in tissue composition and organization that are affected by disease (Sack et al., 2013).

Effects of aging on mechanical properties of brain tissue have been studied with MRE, with several reports of softer tissue in older age (Hiscox et al., 2021). Most investigations have focused age-related differences in stiffness globally or in large regions (Arani et al., 2015; Sack et al., 2009, 2011; Takamura et al., 2020), or local effects in smaller gray matter regions (Delgorio et al., 2021; Hiscox et al., 2018), with an average reported annual decrease in stiffness of 0.8% (or 0.006–0.025 kPa/yr.), and with significant variability in apparent age-related rate of softening depending on the region. However, MRE studies explicitly considering effects of age or age-related neurodegenerative conditions on white matter (WM) mechanical properties have been limited, likely due, in part, to the need for an anisotropic MRE technique to resolve direction-dependent mechanical properties in WM. White matter consists of aligned axon fibers, which cause tissue stiffness to vary depending on the measurement direction. Shear stiffness refers to the resistance of the tissue to deformation, which is typically higher parallel to the fiber direction than perpendicular (Feng et al., 2013). This variation is captured by shear anisotropy, while tensile anisotropy describes the tissue’s resistance to stretching along different directions, with fibers generally offering more resistance along their length (Velardi et al., 2006). In isotropic MRE, which assumes uniform stiffness in all directions, such variations can lead to errors or uncertainty in inversion results (Anderson et al., 2016; McGarry et al., 2021), and capturing directionally-dependent properties is critical for fully understanding how WM mechanics change with age.

Early attempts to characterize mechanical properties in WM tracts assumed mechanical isotropy during inversion (Guo et al., 2013; Johnson et al., 2013), but recently, anisotropic inversion methods have been developed to better capture the directionally-dependent mechanical behavior of WM (McGarry et al., 2021; Romano et al., 2012). These anisotropic methods may vary with respect to the underlying material model, the number of parameters estimated, and the numerical inversion scheme used, with each factor impacting the overall performance of the MRE reconstruction. An early two-parameter approach that models shear modulus parallel and perpendicular to the fiber direction was initially applied to study breast tissue (Sinkus et al., 2005) and skeletal muscle (Green et al., 2013); however, this approach does not capture the potential tensile anisotropy of fibrous tissues. Conversely, an orthotropic, nine-parameter model was applied to WM and the corticospinal tract (Romano et al., 2012, 2014), but estimating the large number of coefficients, especially with different magnitudes, presents a significant challenge using traditional MRE data (Miller et al., 2018a, 2018b). The use of more parameters can introduce increased noise and uncertainty in the estimates, as each parameter may contribute to fitting the noise rather than the true mechanical behavior. In contrast, a three-parameter, nearly incompressible, transversely-isotropic material model has been proposed that includes a substrate shear modulus and two anisotropy parameters representing the differences in Young’s modulus and shear modulus relative to the assumed fiber direction. This model simplifies the problem by focusing on the parameters that have the greatest influence on tissue mechanics (shear modulus, shear anisotropy, and tensile anisotropy), while reducing the likelihood of overfitting. Studies have shown this model sufficiently describes WM mechanics with a minimal number of parameters, avoiding the added complexity and noise sensitivity of larger models (Feng et al., 2013). We have integrated this model into the finite element-based, nonlinear inversion algorithm (NLI) (McGarry et al., 2012)—termed transversely-isotropic NLI (TI-NLI) (McGarry et al., 2021, 2022)—to estimate WM anisotropic properties while also accounting for heterogeneity in both properties and varying fiber direction throughout the brain.

To improve the accuracy and stability of property estimates with TI-NLI, we use multi-excitation MRE to produce diverse displacement data (Anderson et al., 2016; D. R. Smith et al., 2020). Shear waves in fibrous, transversely-isotropic materials travel at different wave speeds based on wave propagation and polarization directions (Tweten et al., 2015), and, as such, MRE displacement data must deform the tissue of interest in multiple directions in order to estimate anisotropic parameters reliably (Tweten et al., 2017). Multi-excitation MRE uses two or more vibration sources, applied sequentially, to generate different wave patterns throughout the brain (D. R. Smith et al., 2020), and, when coupled with TI-NLI, produces repeatable anisotropic property estimates in WM tracts (D. R. Smith et al., 2022).

In this study, we use multi-excitation MRE with TI-NLI to estimate anisotropic mechanical properties of WM tracts of a younger and older population to evaluate and how these parameters are affected by aging. MRE outcomes of substrate shear stiffness, shear anisotropy, and tensile anisotropy are compared between groups of older adults and younger adults. We also investigate how age-related differences in mechanical properties from anisotropic MRE compared with metrics from diffusion MRI, which is commonly used to assess WM structure, and has shown lower fractional anisotropy and higher radial diffusivity in aging (Davis et al., 2009; Lebel et al., 2012; Michielse et al., 2010). As both MRE and diffusion MRI are expected to probe the underlying microstructure of WM, and axonal directions from diffusion MRI are used in the TI-NLI processing, we sought to examine whether anisotropic mechanical property estimates are overly redundant with or influenced by diffusion metrics.

## 2 Methods

### 2.1 Participant information

Participants included 20 younger adults (25.2 ± 2.1 years, 11 females) and 19 older adults (68.1 ± 5.9 years, 8 females). Each participant provided informed, written consent to participate in this study approved by the University of Delaware Institutional Review Board. All participants were screened prior to enrollment to exclude those with any history of neurological disease or brain injury. One older female participant did not complete the entire scan session successfully and this data set was excluded from the study (final older adult group, N = 18, 7 females). Each participant completed an MR imaging protocol on a Siemens 3T Prisma MRI scanner with a 20-channel head coil.

### 2.2 Image acquisition and processing

#### 2.2.1 Multi-excitation MR elastography

**Fig. 1.**
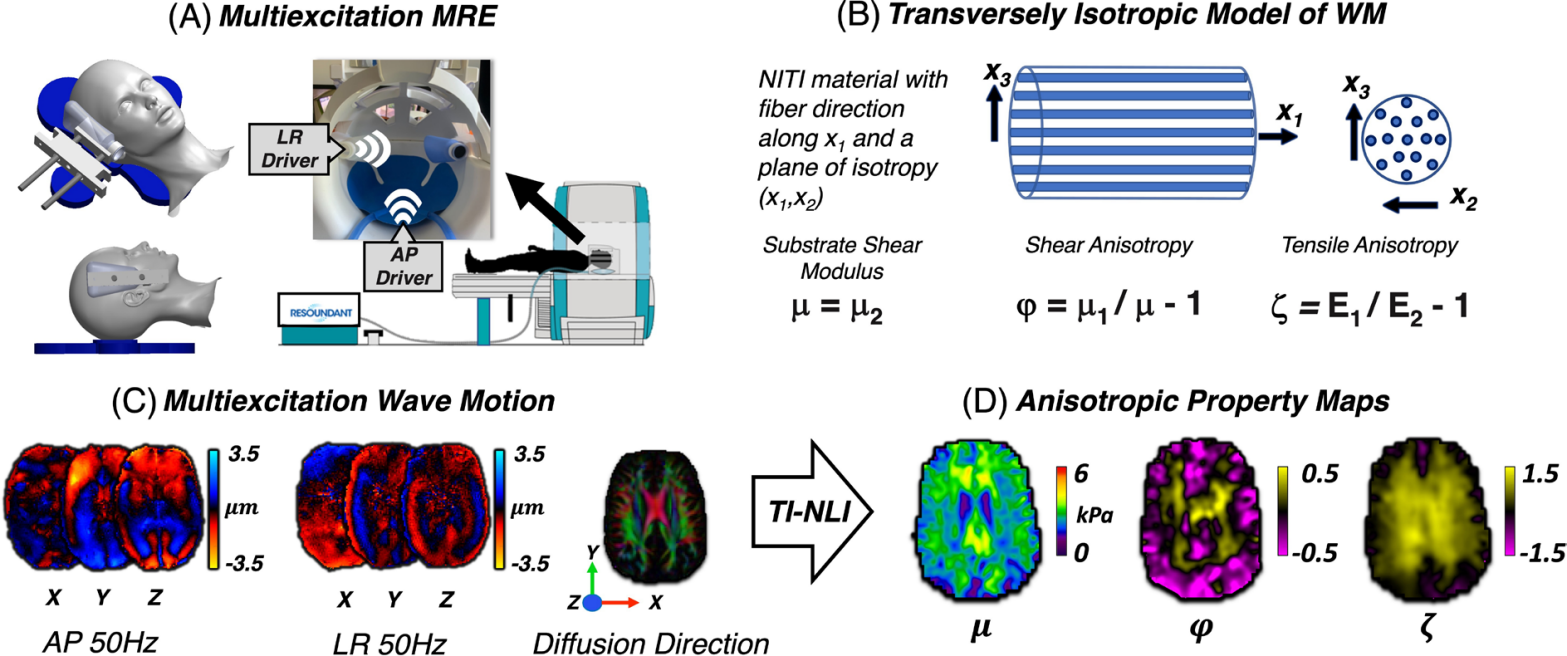
Overview of multi-excitation MRE and TI-NLI. (A) Depiction of the experimental setup showing the custom-designed left-right (LR) bottle actuator, and the anterior-posterior (AP) pillow driver. (B) Schematic of a transversely isotropic material comprising aligned fibers in a substrate, representing fibrous white matter. (C) Inputs to for TI-NLI: AP and LR wave fields at 50 Hz and fiber directions from diffusion MRI. (D) Example anisotropic parameter maps reconstructed with TI-NLI.

Mechanical shear waves were introduced into the brain at 50 Hz using an active pneumatic driver system (Resoundant, Inc., Rochester, MN) with two passive vibration sources: a pillow driver that applies anterior-posterior (AP) motion and a custom-designed left-right (LR) actuator, applied separately, resulting in two distinct, three-dimensional wave fields for inversion (Fig. 1C) (D. R. Smith et al., 2020). Figure 1A illustrates the multi-excitation MRE setup with the passive LR actuator that combines a silicone bottle driver with 3D-printed components that affix the actuator to the head coil to enable stable vibrations and optimal coupling against the participants’ temple (Caban-Rivera et al., 2022; Kailash et al., 2019). Wave fields from both actuations were captured with a 3D multiband, multishot spiral sequence (Johnson et al., 2016; McIlvain, Cerjanic, et al., 2022), that encodes the full vector displacement fields generated in the brain by external actuation. Imaging parameters for each acquisition included 4 phase offsets, 2.0 mm^3^ isotropic voxel resolution, 240 × 240 mm^2^ FOV, 64 slices, and TR/TE = 2240/76 ms. Shear wave displacement fields were calculated via subtraction of opposite-polarity MRE phase images, phase unwrapping was performed using FSL PRELUDE (Jenkinson, 2003), and 50 Hz motion was isolated as the first harmonic from the temporal Fourier transform applied across phase offsets.

#### 2.2.2 Anatomical T1-weighted images

High resolution T1-weighted anatomical images were acquired with an MPRAGE sequence for registration and segmentation with the following imaging parameters: 0.9 mm^3^ isotropic voxels; 256 × 256 mm^2^ FOV; 176 slices; and repetition time (TR)/echo time (TE)/inversion time (TI) = 2300/2.32/900 ms. T1-weighted images were processed using the *fsl_anat* script from FSL (Jenkinson et al., 2012) to automatically segment and register structural images to standard space templates. Images were processed with the following steps: 1) reorientation to MNI standard orientation, 2) bias-field correction with FSL FAST, 3) linear and nonlinear registration to standard space (FLIRT and FNIRT), 4) binary mask created via FNIRT-based brain-extraction, and 5) tissue type segmentation. Bias-corrected, brain-extracted T1-weighted images outputted from *fsl_anat* were registered to MRE images, and the binary mask was applied to the MRE magnitude images prior to calculating the shear wave displacements and estimating mechanical properties.

#### 2.2.3 Diffusion MRI

Diffusion-weighted images were acquired with a simultaneous, multi-slice EPI sequence (210 × 240 × 138 mm^3^ field-of-view, 1.5 mm^3^ resolution, 92 slices, TR/TE = 3520/95.2 ms, b-values = 1500, 3000 s/mm^2^ over 128 directions). Reference scans were acquired with opposite phase encoding direction and zero b-value for distortion corrections using TOPUP (Andersson et al., 2003; S. M. Smith et al., 2004) from FSL. Diffusion images were registered linearly to MRE magnitude images with FSL FLIRT (Jenkinson et al., 2002; Jenkinson & Smith, 2001), and the diffusion gradient directions for each image were rotated accordingly to correct for motion between scans. Diffusion tensors in each voxel were then estimated using FSL Diffusion Toolbox (FDT) which outputs eigenvectors and eigenvalues of the diffusion tensor. To properly fit the multi-shell diffusion data with high b-values, we applied the mean kurtosis fitting option from the dtifit tool in FSL as part of the processing pipeline. From the tensor fitting we acquired multiple DTI metrics: fractional anisotropy (FA), mean diffusivity (MD), radial diffusivity (RD), and mean kurtosis (MK). The first principal eigenvector of the diffusion tensor (the direction of maximal diffusivity) is interpreted as the local fiber axis and used in the inversion algorithm for estimating anisotropic parameters.

### 2.3 Transversely isotropic nonlinear inversion (TI-NLI)

The Transversely Isotropic Nonlinear Inversion is employed to estimate anisotropic mechanical properties based on multi-excitation MRE data. Two distinct wave motion fields, characterized by propagation and polarization directions of shear waves, and the primary eigenvector from diffusion MRI are input to TI-NLI to calculate a set of anisotropic mechanical parameters (McGarry et al., 2021, 2022).

Nonlinear Inversion (NLI) is a finite-element based optimization algorithm that operates in two step. First, the forward problem estimates displacement fields based on the material properties of the tissue. Second, the inverse problem iteratively updates the unknown material properties by minimizing the error between the measured and calculated displacements (McGarry et al., 2012; Van Houten et al., 1999).

In this implementation, TI-NLI uses a nearly incompressible, transversely isotropic model, which accounts for the directional stiffness of fibrous tissue, such as the brain WM. This model assumes that tissue stiffness varies depending on the direction of the fibers, which is critical for understanding how mechanical properties behave along different orientations within the brain (Feng et al., 2013; Tweten et al., 2015). The outputs from TI-NLI include spatial maps of three key parameters (Fig. 1D):

$$\begin{array}{c} \text{Substrate}\;\text{Shear}\;\text{Modulus}:G=G_{2}\\ \;\;\;\;\;\;\;\text{Shear}\;\text{Anisotropy}:\varphi =\frac{G_{1}}{G_{2}}-1\\ \;\;\;\;\;\;\text{Tensile}\;\text{Anisotropy}:\zeta =\frac{E_{1}}{E_{2}}-1 \end{array}$$

Here, G refers to the complex valued substrate shear modulus and Young’s modulus is denoted by E, while subscripts 1 and 2 refer to components parallel and perpendicular to the fiber axis, respectively. G is complex such that $\text{G}={\text{G}}^{\prime }+\text{i}{\text{G}}^{\prime \prime }$ , where ${\text{G}}^{\prime }$ is the storage modulus, or the real component of the shear modulus describing elastic behavior of tissue, and ${\text{G}}^{\prime \prime }$ is the loss modulus, or the imaginary component of the shear modulus describing viscous behavior of the tissue. Additionally, we compute substrate shear stiffness $\mu =\mu_{2}=\frac{2{\left|G\right|}^{2}}{G^{\prime }+\left|G\right|}$ as a composite parameter of the substrate shear modulus which is proportional to the square of the wave speed in a viscoelastic material and is a measure commonly reported in MRE literature (Manduca et al., 2021). For the TI-NLI inversion, we solve for isotropic damping with real-valued φ and ζ, such that both real and imaginary components of tissue behavior are assumed to exhibit the same anisotropy (Jyoti et al., 2023). Additionally, we report an effective stiffness in the fiber $\mu_{1}=\;\mu_{2}\left(1+\phi \right)$, estimated using the shear anisotropy, described as the shear stiffness parallel to the fiber direction (D. R. Smith et al., 2023).

We also performed an isotropic inversion using the traditional NLI for comparison, assuming the shear stiffness does not depend upon a given fiber axis. This was performed using just the 50 Hz AP wavefield for each subject to determine the isotropic shear stiffness, $\mu_{\text{iso}}$ . Our primary outcomes were $\mu_{2}$, ϕ, and ζ, but we repeated analyses for $\mu_{1}$ and $\mu_{iso}$ to aid our interpretation of any observed differences in shear anisotropy or shear stiffness between younger and older adults.

### 2.4 Tract-based spatial statistics

We investigated age-based differences in WM mechanical properties through a voxel-wise analysis using tract-based spatial statistics (TBSS) (S. M. Smith et al., 2006). TBSS accounts for alignment inconsistencies across subjects that can confound voxel-wise statistics by generating a common white matter skeleton for voxel-wise analysis between subjects. FA maps from diffusion MRI underwent preprocessing, including erosion, removal of outlier slices, and nonlinear registration to an average FA template (*FMRIB58_FA*) in standard space at 1 mm isotropic resolution, with voxels in which FA > 0.2 were included. The main outcome from TBSS is the registration of all subjects’ data to an existent mean FA skeleton of the white matter, onto which the subject-specific FA data were projected. Reconstructed MRE property maps ($\mu_{2}$, $\mu_{1}$, $\mu_{\text{iso}}$ , ϕ, ζ), in the same native space as FA, were then projected onto the mean FA skeleton using *tbss_non_fa* in FSL for statistical analyses in standard space. Additionally, we included other diffusion metrics mean diffusivity (MD), radial diffusivity (RD), and mean kurtosis (MK) to the analysis. Voxel-wise statistics were performed with FSL *randomize* including two sample t-tests for all mechanical parameters, yielding statistical maps comparing younger adults with older adults at significance level α=0.05 . We calculated the percentage of voxels in the skeleton that exhibited significant differences between older and younger adults. We also compared the statistical maps for each of the MRE measures and FA from diffusion, calculating overlap in significant voxels describing differences between age groups.

### 2.5 ROI-based analysis of WM tracts

We performed region-of-interest (ROI) analyses using WM tract masks to confirm the TBSS findings. Tract masks were obtained from white matter atlases (Hua et al., 2008; Mori et al., 2008) and applied to the TBSS outputs in standard space. Tracts of interest included: corpus callosum body (CCB), forceps major (Fmaj), forceps minor (Fmin), corona radiata (CR), corticospinal tract (CST), anterior thalamic radiation (ATR), posterior thalamic radiation (PTR), and superior longitudinal fasciculus (SLF). A visualization of mean FA map and the white matter skeleton overlaid by the 8 WM tract ROIs is shown in Supplementary Figure S1. Average property values were calculated within each tract mask for voxels that coincide with the WM skeleton from TBSS. Two-sample t-tests compared properties between younger and older adults for each tract with Bonferroni correction for multiple comparisons. We additionally employed a logistic regression analysis to determine the distinct contribution of anisotropic MRE parameters relative to diffusion MRI measures in classifying age groups within each ROI. Specifically, we first included each diffusion MRI parameter—Fractional Anisotropy (FA), Mean Diffusivity (MD), Radial Diffusivity (RD), and Mean Kurtosis (MK)—individually as classifiers in the model. Following this, we assessed the added value of each MRE parameter—$\mu_{2}$, $\mu_{1}$, $\mu_{\text{iso}}$ , ϕ, ζ—by incorporating them separately into the model. This approach allowed us to evaluate whether the MRE parameters provide additional information for classifying age groups that are not captured by diffusion MRI metrics alone.

## 3 Results

**Fig. 2.**
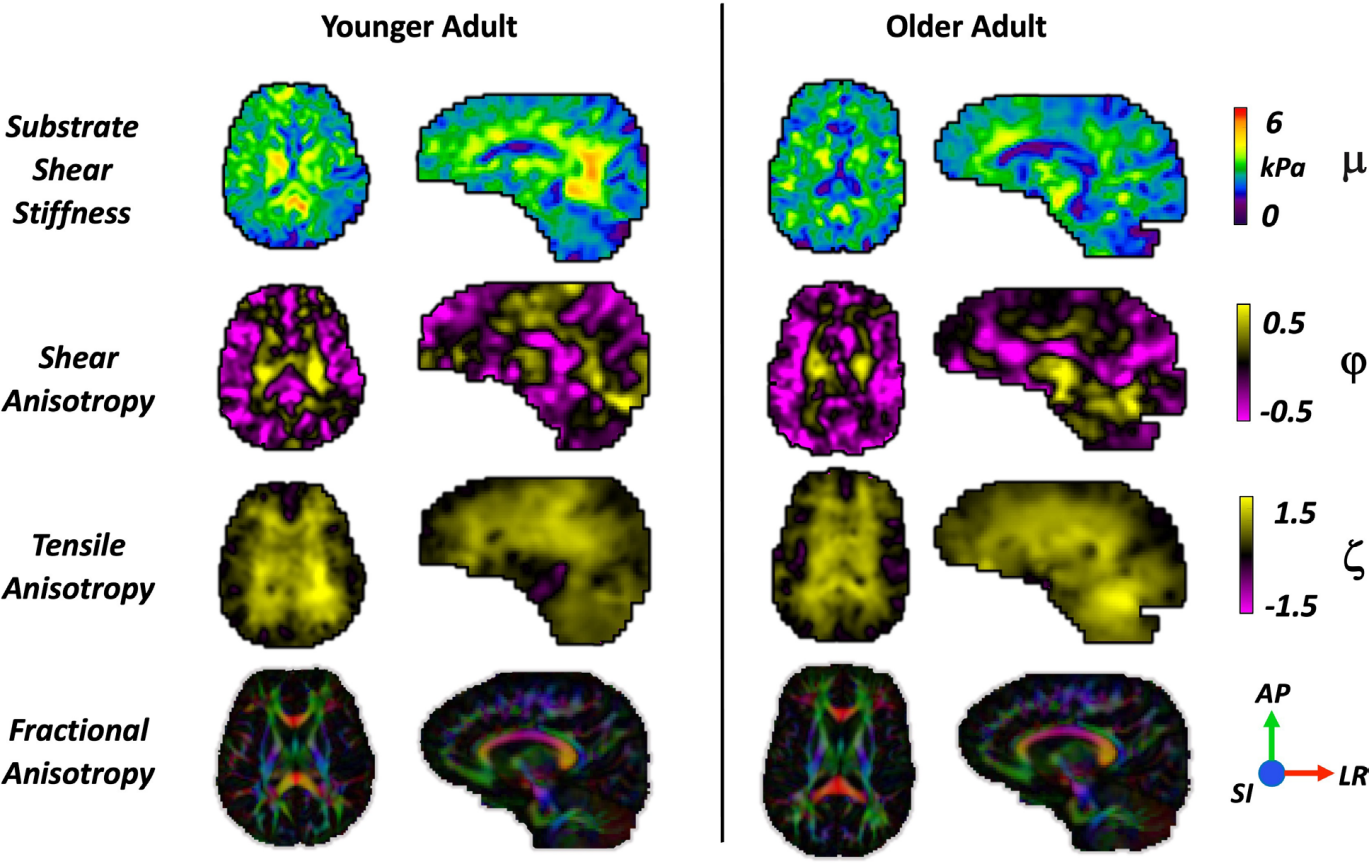
Comparison of representative whole-brain property maps between one younger adult (30 years, F) and one older adult (61 years, F). Rows 1–3 illustrate outputs from the transversely isotropic nonlinear inversion, where anisotropic parameters have similar structural contrast between the groups, but lower values across the whole brain in the older adults. The 4th row shows the colored fractional anisotropy map estimated from the diffusion tensor with the corresponding direction-color combinations: x-red, y-green, and z-blue. We observed lower values for global stiffness, anisotropy, and FA in the older adults.

Representative property maps for one younger adult (30 years, female) and one older adult (61 years, female) are shown in Figure 2 for qualitative comparison of the anisotropic mechanical properties and their distribution in axial and sagittal slices. In general, the younger adult shows higher whole-brain substrate stiffness, while shear anisotropy and tensile anisotropy show similar structure between ages, with the older adult exhibiting lower anisotropy in the WM regions. Table 1 provides a summary of the findings for the anisotropic MRE parameters with mean, standard deviation, and range for each WM tract.

**Table 1.** Overview of TI-NLI property means and standard deviations in white matter tracts for young adults (YA) versus older adults (OA).

Region	µ_2_, YA (kPa)	µ_2_, OA (kPa)	µ_2_, p-value	φ, YA	φ, OA	φ, p-value	ζ, YA	ζ, OA	ζ, p-value
CCB	2.47 ± 0.29 (2.01–3.13)	2.47 ± 0.35 (1.86–3.29)	p = 0.974	0.25 ± 0.10 (0.04–0.47)	0.18 ± 0.09 (0.06–0.38)	p = 0.015	1.11 ± 0.16 (0.80–1.38)	0.83 ± 0.13 (0.52–1.08)	**p** **<** 0.001**
FMAJ	2.74 ± 0.20 (2.42–3.06)	2.62 ± 0.19 (2.24–2.89)	p = 0.068	0.01 ± 0.09 (-0.11–0.17)	-0.07 ± 0.12 (-0.28–0.21)	p = 0.033	1.10 ± 0.19 (0.45–1.42)	0.96 ± 0.13 (0.70–1.24)	p = 0.012
FMIN	2.94 ± 0.23 (2.61–3.50)	2.61 ± 0.23 (2.30–3.22)	**p** **<** 0.001**	0.05 ± 0.05 (-0.04–0.15)	-0.06 ± 0.07 (-0.20–0.06)	**p** **<** 0.001**	0.88 ± 0.20 (0.52–1.23)	0.53 ± 0.20 (0.17–0.88)	**p** **<** 0.001**
CR	2.71 ± 0.12 (2.58–3.02)	2.61 ± 0.17 (2.42–3.05)	p = 0.057	0.19 ± 0.03 (0.13–0.25)	0.10 ± 0.05 (-0.01–0.17)	**p** **<** 0.001**	1.05 ± 0.13 (0.74–1.26)	0.76 ± 0.12 (0.45–0.93)	**p** **<** 0.001**
CST	2.79 ± 0.22 (2.45–3.42)	2.73 ± 0.22 (2.38–3.21)	p = 0.371	0.35 ± 0.07 (0.18–0.42)	0.25 ± 0.06 (0.14–0.34)	**p** **<** 0.001**	1.13 ± 0.13 (0.85–1.32)	0.88 ± 0.14 (0.59–1.09)	**p** **<** 0.001**
ATR	3.00 ± 0.15 (2.80–3.33)	2.78 ± 0.21 (2.49–3.24)	**p** **=** 0.001*	0.09 ± 0.03 (0.04–0.15)	0.04 ± 0.04 (-0.07–0.08)	**p** **<** 0.001**	1.01 ± 0.16 (0.72–1.25)	0.75 ± 0.13 (0.47–0.94)	**p** **<** 0.001**
PTR	2.86 ± 0.17 (2.62–3.27)	2.73 ± 0.20 (2.39–3.05)	p = 0.040	-0.04 ± 0.10 (-0.21–0.10)	-0.07 ± 0.07 (-0.20–0.08)	p = 0.303	1.17 ± 0.19 (0.44–1.42)	1.01 ± 0.09 (0.86–1.18)	**p** **=** 0.003*
SLF	2.72 ± 0.13 (2.57–3.02)	2.67 ± 0.15 (2.49–2.98)	p = 0.284	0.10 ± 0.03 (0.03–0.14)	0.02 ± 0.04 (-0.04–0.09)	**p** **<** 0.001**	1.02 ± 0.14 (0.61–1.18)	0.79 ± 0.09 (0.55–0.91)	**p** **<** 0.001**

Statistically significant group differences from Students t-tests after Bonferroni correction are bolded and starred (*) for significance level *p < 0.00625, **p < 0.001.

Data quality of the AP and LR excitations was assessed via an octahedral shear strain-based SNR (OSS-SNR) to ensure sufficient data quality for stable inversion (McGarry et al., 2011). Supplementary Figure S2 shows the OSS-SNR for both excitation directions separated for younger and older adults. Data from all AP and LR excitations met the minimum OSS-SNR threshold of 3.0 to ensure stability of the inversion (Hannum et al., 2022; McGarry et al., 2011), with no significant differences in OSS-SNR between YA and OA groups for either AP (YA: 10.78 ± 3.39, OA: 9.55 ± 1.45; p = 0.171) or LR (YA: 7.21 ± 2.39, OA: 6.83 ± 2.50; p = 0.633) exictation.

### 3.1 Shear stiffness (μ)

**Fig. 3.**
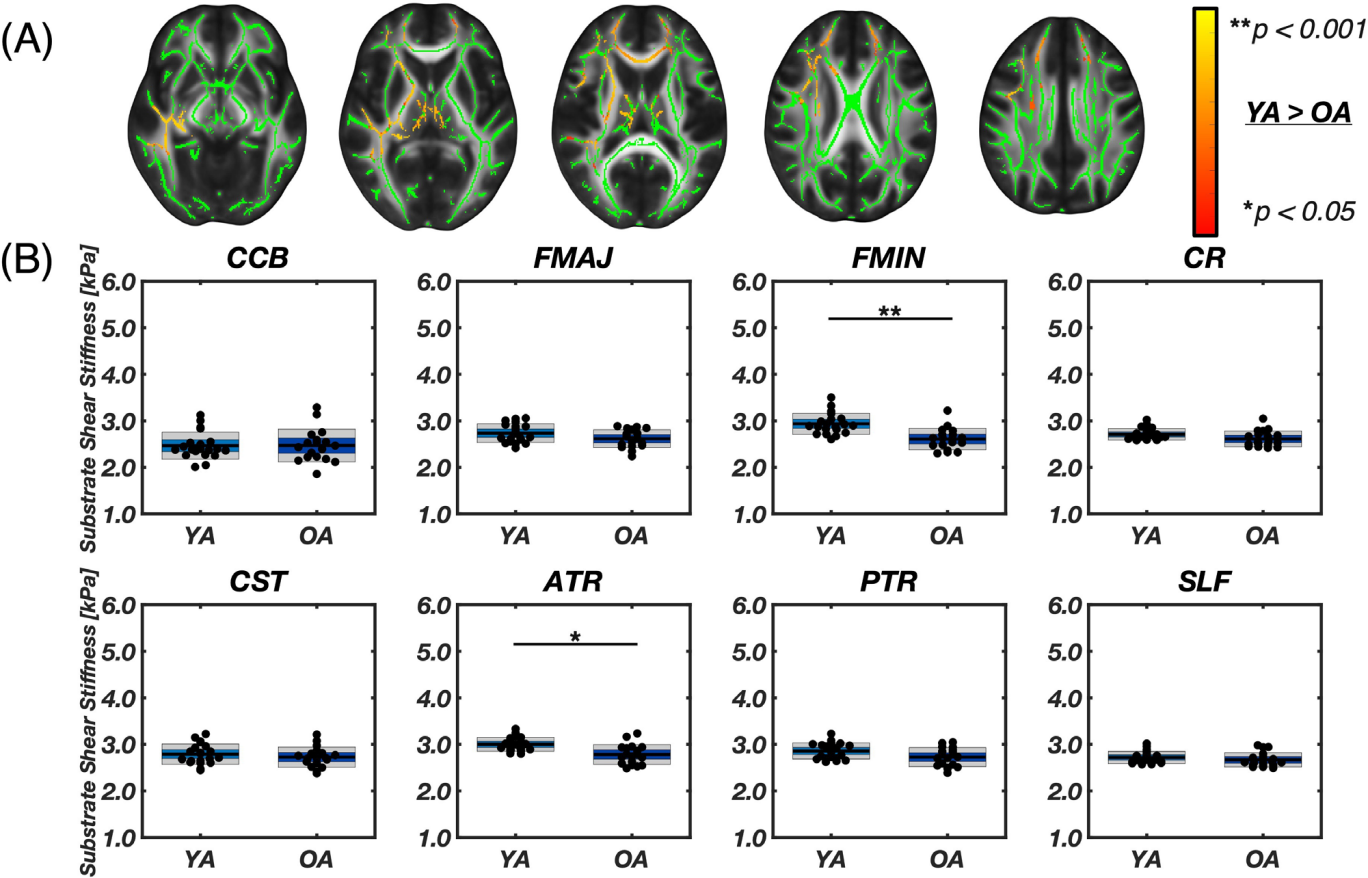
(A) Results from voxel-wise analysis using tract based spatial statistics (TBSS) for axial slices [66, 77, 86, 98, 107], showing the white matter skeleton mask in green and the contrast of Young Adult > Older Adult for substrate shear stiffness (µ_2_) overlaid with the red-yellow color bar, which represents voxel-wise p-values between 0.05–0.001. No significant voxels were found in the OA > YA contrast. (B) Segmented white matter region averages of µ_2_. Asterisks (*) indicate significant differences between groups as determined by two-sample Student’s t-tests with Bonferroni correction (*p < 0.00625, **p < 0.001). Older adults showed significantly lower µ_2_ in the forceps minor (FMIN, p < 0.001**), and anterior thalamic radiation (ATR, p = 0.0011*).

Figure 3 presents differences in substrate shear stiffness ($\mu_{2}$) between groups. Through voxel-wise analysis with TBSS, we observed small clusters in the middle to anterior regions (right hemisphere) where $\mu_{2}$ was significantly lower in older adults compared to younger adults (11.3% of the WM skeleton). Through ROI analysis, we observed that the older adults group exhibited significantly lower stiffness in the Fmin (2.61 ± 0.23 vs. 2.94 ± 0.23 kPa; -11.8%; p < 0.001) and in the ATR (2.78 vs. 3.00 kPa; p < 0.0011), with the latter having the highest $\mu_{2}$ in both groups. We observed the largest difference in $\mu_{2}$ between younger and older adults in the Fmin at 11.8% ($\Delta \mu_{2\text{abs}}$ = 0.328 kPa; p < 0.001) and the smallest was seen in the CCB at 0.14% ($\Delta \mu_{2\text{abs}}$ = 0.003 kPa; p = 0.974). No voxels from TBSS or ROIs appeared significantly stiffer in older adults compared to younger adults. Interestingly, the parallel shear stiffness ($\mu_{1}$) had many more significantly different voxels throughout the white matter skeleton (49.70%), with all ROIs except the CCB and PTR having significantly lower $\mu_{1}$ in older adults, demonstrating that parallel and perpendicular stiffness may have distinct sensitivity with respect to aging in white matter. The highest parallel shear stiffness was observed in the CST—for younger (3.91 ± 0.42 kPa) and older adults (3.49 ± 0.45 kPa), and the largest difference in $\mu_{1}$ was seen in the Fmin at 22.3% ($\Delta \mu_{1\text{abs}}$ = 0.640 kPa; p < 0.001) and smallest in the CCB at 6.0% ($\Delta \mu_{1\text{abs}}$ = 0.184 kPa; p = 0.271). Although both $\mu_{1}$and $\mu_{2}$ showed the highest differences between young and older adults in the same tract (Fmin), the age effect on the parallel stiffness is much larger than on the perpendicular stiffness. For isotropic shear stiffness ($\mu_{\text{iso}}$ ), 47.67% of the skeleton voxels were significant and seemed more comparable to the parallel stiffness with widespread reduction with age in WM. TBSS and ROI analysis results are plotted in Supplementary Figures S3 for $\mu_{1}$ and S4 for $\mu_{\text{iso}}$ , with Supplementary Table S1 summarizing both parameters’ outcomes for all eight tracts. Supplementary Figures S5 and S6 show the segmented WM ROI averages of $\mu_{2}$, $\mu_{1}$, and $\mu_{\text{iso}}$ , side-by-side and separated for younger and older adults.

### 3.2 Shear anisotropy (ϕ)

**Fig. 4.**
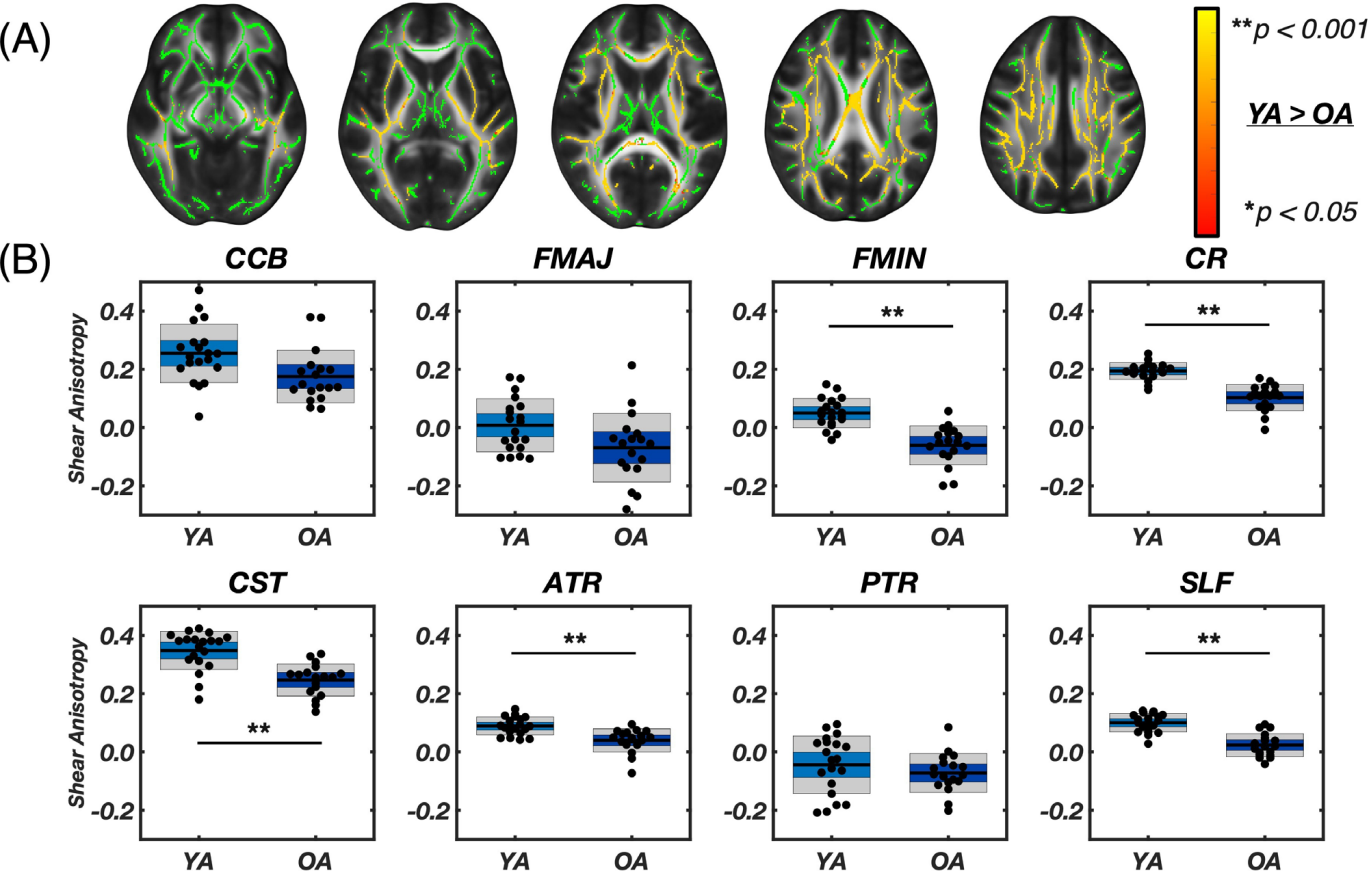
(A) Results from voxel-wise analysis using tract based spatial statistics (TBSS) for axial slices [66, 77, 86, 98, 107], including the white matter skeleton mask in green and the contrast of Young Adult > Older Adult for shear anisotropy (φ) overlaid by the red-yellow color bar, which represents voxel-wise p-values between 0.05–0.001. The OA > YA analysis contrast did not result in significant voxels. (B) Segmented white matter region averages of φ. Asterisks (*) indicate significant differences between groups as determined by two-sample Student’s t-tests with Bonferroni correction (* p < 0.00625, ** p < 0.001). Older adults had significantly lower φ in multiple tracts: Fmin (p < 0.001**), CR (p < 0.001**), CST (p < 0.001**), ATR (p < 0.001**), and SLF (p < 0.001**).

Figure 4 presents differences in shear anisotropy between groups. TBSS analysis showed many voxels in WM, particularly in the corpus callosum and periventricular regions, exhibited lower shear anisotropy in older adults (39.30%). Lower shear anisotropy indicates that the ratio of shear stiffness parallel to fiber direction relative to the stiffness perpendicular to fibers is smaller. Since most tracts exhibit a positive ϕ in tracts in younger adults, lower anisotropy in older adults indicates that the stiffness parallel to fibers is more similar to the perpendicular stiffness with age. Most tract ROIs showed significantly lower ϕ in older adults versus younger adults except the CCB (0.18 ± 0.09 [range: 0.06–0.38] vs. 0.25 ± 0.10 [range: 0.04–0.47]; p = 0.015), Fmaj (-0.07 ± 0.12 [range: -0.28–0.21] vs. 0.01 ± 0.09 [range: -0.11–0.17]; p = 0.033), and PTR (-0.07 ± 0.07 [range: -0.20–0.08] vs. -0.04 ± 0.10 [range: -0.21–0.10]; p = 0.303). The negative shear anisotropy observed in the PTR and Fmaj suggests that, in these fibers, the material exhibits less resistance to shearing along the fibers compared to shearing perpendicular to them. The highest shear anisotropy was seen in the CST for both groups—0.35 ± 0.07 in younger adults and 0.25 ± 0.06 in older adults—whereas the absolute difference between groups was largest in the Fmin [$\Delta \phi_{\text{abs}}$ = 0.11; p < 0.001] and smallest in the PTR [$\Delta \phi_{\text{abs}}$ = 0.03; p = 0.303]. No voxels from TBSS or ROIs appeared to have significantly higher shear anisotropy in older adults compared to younger adults.

### 3.3 Tensile anisotropy (ζ)

**Fig. 5.**
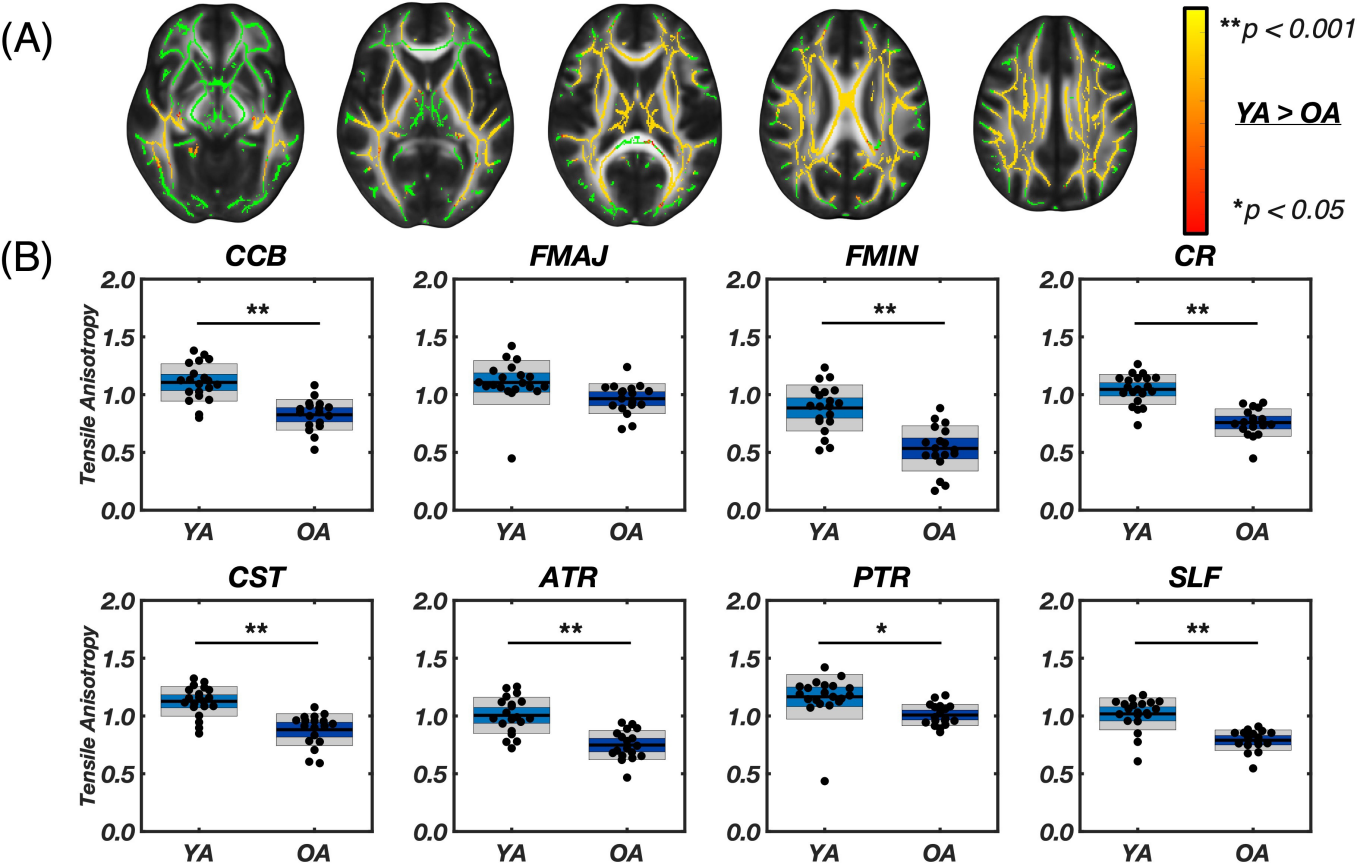
(A) Results from voxel-wise analysis using tract based spatial statistics (TBSS) for axial slices [66, 77, 86, 98, 107], including the white matter skeleton mask in green and the contrast of Young Adult > Older Adult for tensile anisotropy (ζ) overlaid by the red-yellow color bar, representing voxel-wise pvalues between 0.05–0.001. The OA > YA analysis contrast did not result in significant voxels. (B) Segmented white matter region averages of ζ. Asterisks (*) indicate significant differences between groups as determined by two-sample Student’s t-tests with Bonferroni correction (* p < 0.00625, ** p < 0.001). Older adults had significantly lower ζ in most tracts: CCB (p < 0.001*), Fmin (p < 0.001**), CR (p < 0.001**), CST (p < 0.001**), ATR (p < 0.001**), PTR (p = 0.0029*), and SLF (p < 0.001**).

Figure 5 presents differences in tensile anisotropy between groups. TBSS analysis showed that most voxels of the cerebrum exhibited lower ζ in older adults (56.94% of total skeleton). Older adults exhibited lower μ in all WM tract ROIs investigated (all p < 0.007) except the Fmaj (0.96 ± 0.13 [range: 0.70–1.24] vs. 1.10 ± 0.19 [range: 0.45–0.1.42]; p = 0.012), with absolute differences in anisotropy between younger and older adults being the smallest in the Fmaj at 13.5% ($\Delta \zeta_{\text{abs}}$ = 0.14; p = 0.012) and largest in the Fmin at 49.34% ($\Delta \zeta_{\text{abs}}$ = 0.35; p < 0.001). Tensile anisotropy in WM tracts in younger adults is near 1, indicating that the tensile modulus is approximately double in the fiber direction (D. R. Smith et al., 2022); lower ζ indicates tensile modulus in the fiber direction is lower in older adults. As with stiffness and shear anisotropy, no voxels from TBSS or ROIs appeared to have significantly higher tensile anisotropy in older adults compared to younger adults.

### 3.4 Contrasting MRE and DTI parameters using TBSS

**Fig. 6.**
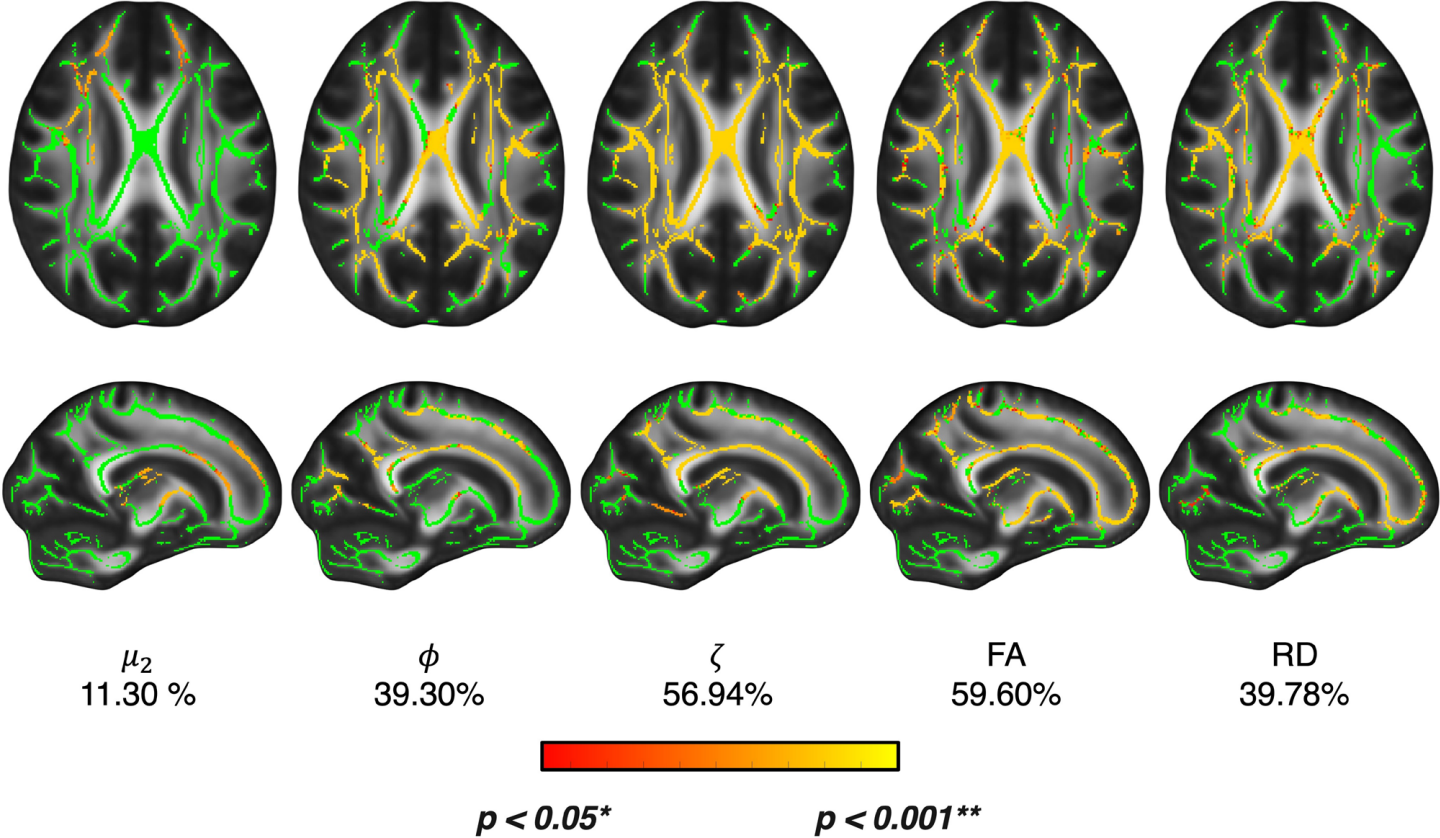
Summary of TBSS voxel-wise statistical analysis for fractional anisotropy (FA) and radial diffusivity (RD) and MRE parameters substrate shear stiffness—µ!, shear anisotropy—φ, and tensile anisotropy—ζ comparing young and older adults. The images show axial and sagittal slices (of the right hemisphere) in which we observe the mean fractional anisotropy atlas image in grayscale, the white matter skeleton mask in green, overlaid with a red-yellow colormap indicating the significant voxels with p values between 0.05–0.01. Substrate shear stiffness has the smallest percentage of significant voxels within the cerebrum white matter, whereas the shear anisotropy and tensile anisotropy have comparable percentages to FA and RD respectively.

Supplementary Figures S7–S10 show the results from TBSS and ROI analyses for FA, MD, RD, and MK respectively, and Supplementary Table S2 provides the mean, standard deviation, and range for each ROI. In addition to evaluating the individual MRE and DTI parameters, we contrasted the anisotropic MRE measures with diffusion tensor imaging (DTI) parameters to investigate potential overlap and differences in their sensitivity to age-related changes in white matter (Fig. 6). The percentage of significant voxels exhibiting age-related differences (Younger Adult > Older Adult, or Older Adult > Younger adult; p < 0.05) was calculated for each parameter across the white matter skeleton of the cerebrum. Fractional anisotropy (FA) from DTI (59.60%) had the highest percentage of significant voxels in the skeleton, with tensile anisotropy (ζ) in close second (56.94%), followed by radial diffusivity (RD) (39.78%) and shear anisotropy (ϕ) (39.30%), and substrate shear stiffness (μ) (11.30%). A side-by-side comparison of the same axial slice for all 9 parameters investigated in this study is provided in Supplementary Figure S11.

**Fig. 7.**
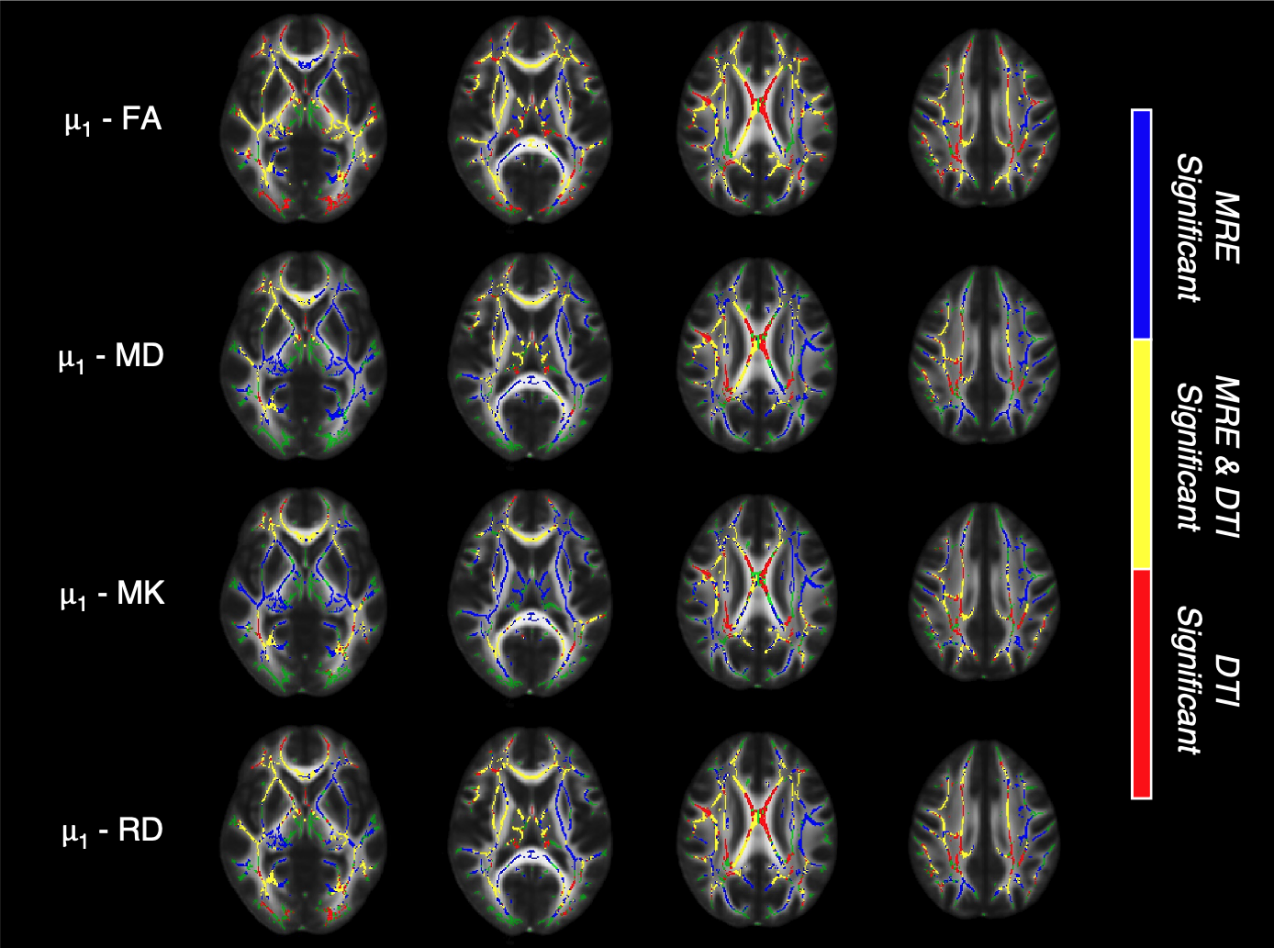
Comparison of areas with significant voxels resulting from contrasting younger versus older adults, between MRE parameter μ1 and DTI parameters FA, MD, MK, and RD. The background image is the mean fractional anisotropy atlas overlaid by the white matter skeleton mask in green. Three colors are shown to distinguish 1) voxels in which μ1 is significant only, blue, 2) voxels in which DTI parameters are significant only, red, and 3) both μ_1_ and DTI parameters are significant (where they overlap), yellow. Areas where neither MRE nor DTI parameters are significant and do not have a color overlaid over the mask and appear as green.

To further investigate the relationship between MRE ($\mu_{2}$, $\mu_{1}$, $\mu_{\text{iso}}$ , ϕ, ζ) and DTI measures (FA, RD, MD, MK), we determined 1) voxels where MRE parameters are significant; 2) voxels where DTI parameters are significant, and 3) voxels where both parameters are significant or neither. For each MRE parameter we compared against each DTI parameter. Out of the three shear stiffness metrics ($\mu_{2}$, $\mu_{1}$, $\mu_{\text{iso}}$ ), $\mu_{1}$ had the greatest number of voxels that were significant without overlapping with the DTI parameters shown in Figure 7. Comparisons for $\mu_{2}$ and $\mu_{\text{iso}}$ with DTI parameters are shown in Supplementary Figures S12 and S13, respectively. In Supplementary Figures S14 and S15, we observe that ϕ and ζ have voxels spread through the skeleton that are significant without overlap with the DTI parameters. With widespread differences in significant voxels throughout the skeleton, our results suggest that the age-related effects on anisotropic MRE parameters may be present in distinct regions compared to DTI-derived measures of diffusion anisotropy.

We performed a logistic regression analysis to determine the added value of MRE anisotropy parameters beyond DTI-derived FA and RD in classifying between age groups. As shown in Tables 2 and 3, both shear anisotropy and tensile anisotropy exhibited significant effects in several tracts when added to the model after FA and/or RD, indicating that these MRE measures, indeed, provide complementary information to DTI metrics in discriminating between younger and older adults. For additional DTI metrics MD and MK, the outputs are shown in Supplementary Tables S3 and S4, with similar results. Overall, these findings suggest that anisotropic MRE parameters, particularly shear and tensile anisotropy, are sensitive to age-related changes in white matter and may contribute distinct information from that of diffusion metrics.

**Table 2.** Logistic regression results summarized by p-values and β values, for each of the MRE parameters separated by columns, which were added to the regression after fractional anisotropy (FA) to classify younger versus older adults.

Region	µ_2_, p-value	µ_2_, β	φ, p-value	φ, β	ζ, p-value	ζ, β	µ_iso_, p-value	µ_iso_, β	µ_1_, p-value	µ_1_, β
CCB	0.571	0.001	0.013	14.247	**0.006** *	18.382	0.056	0.004	0.054	0.002
Fmaj	0.196	0.002	0.032	9.129	0.056	5.698	0.044	0.003	0.013	0.004
Fmin	**0.006** *	0.007	**0.003** *	38.960	**0.004** *	9.446	**0.005** *	0.009	0.007	0.010
CR	0.121	0.004	**0.005** *	79.236	**0.004** *	18.004	0.024	0.030	**0.005** *	0.006
CST	0.419	0.002	**0.004** *	27.527	0.011	25.665	0.021	0.013	0.038	0.002
ATR	0.015	0.007	0.009	114.662	**0.006** *	13.719	0.081	0.039	**0.005** *	0.007
PTR	0.076	0.004	0.127	7.363	0.024	7.779	0.047	0.003	0.048	0.003
SLF	0.438	0.002	**0.005** *	57.820	**0.003** *	16.554	0.012	0.013	**0.005** *	0.006

The rows of the table depict the different white matter tract ROIs investigated in the study. Statistically significant effects are bolded and starred (*)

**Table 3.** Logistic regression results summarized by p-values and β values, for each of the MRE parameters separated by columns, which were added to the regression after mean diffusivity (MD) to classify younger versus older adults.

Region	µ_2_, p-value	µ_2_, β	φ, p-value	φ, β	ζ, p-value	ζ, β	µ_iso_, p-value	µ_iso_, β	µ_1_, p-value	µ_1_, β
CCB	0.979	0.000	0.036	11.385	0.014	21.637	0.105	0.003	0.269	0.001
Fmaj	0.257	0.002	0.024	10.116	0.068	5.399	0.053	0.003	0.014	0.004
Fmin	0.010	0.006	0.008	36.074	0.008	10.761	0.008	0.009	0.011	0.009
CR	0.106	0.005	**0.004** *	77.666	**0.004** *	17.552	0.018	0.027	**0.006** *	0.006
CST	0.333	0.002	**0.003** *	25.895	**0.004** *	19.544	0.019	0.010	0.033	0.002
ATR	0.016	0.006	**0.006** *	97.990	**0.004** *	14.106	0.091	0.048	**0.004** *	0.007
PTR	0.040	0.005	0.106	7.900	0.027	7.640	0.033	0.003	0.031	0.003
SLF	0.350	0.003	**0.003** *	55.936	**0.002** *	15.132	0.012	0.013	**0.003** *	0.007

The rows of the table depict the different white matter tract ROIs investigated in the study. Statistically significant effects are bolded and starred (*)

## 4 Discussion

This paper presents a cross-sectional study aimed at establishing a baseline of anisotropic parameters in the aging brain through application of anisotropic MRE with transversely isotropic nonlinear inversion. Our investigation focuses on a group of normal older adults, and we compare the substrate shear stiffness, shear anisotropy, and tensile anisotropy between younger and older adults using wave fields acquired via multi-excitation MRE. Analysis of mechanical properties in the two groups revealed significant differences in several white matter regions, with the older group exhibiting slightly lower stiffness than the younger group but much lower mechanical anisotropy, likely reflecting altered WM structure in older adults. Notably, our results show that voxels that are significantly different between young and older adults are unique between MRE and DTI measures in many areas of the WM skeleton, particularly for the anisotropy terms and the parallel shear stiffness. These findings are further supported by logistic regression results which demonstrate that MRE parameters contribute unique information when characterizing WM properties in aging.

Despite the commonly reported lower tissue stiffness in age, we observed a 4.57% lower substrate shear stiffness $\mu_{2}\;$ averaged across all tracts in older adults compared to their younger counterparts, with equivalent annual differences between 0 and -0.0076 kPa/year. Only a small percentage of voxels from TBSS analysis of stiffness were significantly lower in older adults, and only the forceps minor and anterior thalamic radiation had statistically significantly lower $\mu_{2}$. This lower stiffness appears less pronounced than previous studies employing isotropic material models in global or gray matter regions, with reported decreases in cerebral stiffness ranging from -0.008 to -0.025 kPa/year (Arani et al., 2015; Lv et al., 2020; Sack et al., 2009; Takamura et al., 2020). Using isotropic nonlinear inversion methods, Delgorio et al. (2021) reported an annual decrease in hippocampal stiffness of -0.014 kPa/year, while Hiscox et al. (2018) reported -0.006 kPa/year, with up to 24% difference between younger and older groups in specific subcortical regions.

Compared to stiffness perpendicular to the fibers, age-related effects on mechanical anisotropy were larger. Across white matter tracts, older adults showed lower shear anisotropy and tensile anisotropy, with ζ exhibiting larger differences between groups. In tracts with higher anisotropy, the differences in ϕ_YA-OA_ = 0.1 and ϕ_YA-OA_ = 0.07 for the CST and CCB respectively. In both tracts, this represents an ≈28% difference in shear anisotropy, whereas the substrate shear modulus in these tracts changed by <3%. Interpreted in terms of directional stiffness, the CST μ_1_/μ_2_ ratio (1 + ϕ) decreased from 1.35 to 1.25, corresponding to shear modulus parallel to the fibers (μ_1_) being ≈0.42 kPa (~11%) lower in older adults (3.91 vs. 3.49 kPa), while μ_2_ was only ≈2% lower. These tract-level examples illustrate that age-related changes are preferentially expressed in anisotropic mechanical properties and stiffness along the fibers, whereas the underlying substrate shear stiffness is comparatively preserved. Interestingly, while there were minor differences between age groups in substrate stiffness, the TBSS results suggest that there are potentially asymmetric hemispheric effects, with the left side of the brain showing a greater age-related difference in substrate stiffness. It is unlikely that this is an artifact from actuator placement, as waves propagate from many points across the skull to deform both sides of the brain evenly (D. R. Smith et al., 2020), and the results instead may be consistent with hemispheric age-related differences observed in other imaging studies (Bennett et al., 2010).

The diminished anisotropy likely explains the discrepancy between only moderately lower substrate shear stiffness in our study compared to larger aging effects in previous studies employing isotropic models. Considered differently, stiffness *perpendicular* to the fibers was not strongly affected by age, but stiffness *parallel* to the fibers was significantly lower in the older group, in general, and becomes more similar to perpendicular stiffness. Isotropic inversions of anisotropic tissue return an effective shear modulus that depends on the orientation of wave propagation and polarization relative to the fiber architecture, and can be biased toward the stiffer direction in strongly anisotropic tracts (Anderson et al., 2016; D. R. Smith et al., 2020; Tweten et al., 2017). The marked loss of anisotropy and parallel stiffness observed here may, therefore, help reconcile the modest age-related changes in μ_2_ with the larger stiffness differences reported in earlier isotropic brain MRE studies.

The specific neurobiological interpretation of anisotropic mechanical properties in the brain and associated effects of age is not known. Tensile stiffness along the fiber direction, as reflected by tensile anisotropy (ζ), is likely most closely related to the condition of axonal fibers themselves, where disruptions such as axonal degeneration could lead to observed reductions in stiffness with age. However, shear stiffness and shear anisotropy (ϕ) may more strongly arise from the extra-axonal matrix, including components such as the oligodendrocyte network and myelin sheath, which are crucial for maintaining the structure and function of WM, and allow the axons to be integrated in a network as a material. Lower shear anisotropy in older age may be consistent with increased RD—suggestive of demyelination and oligodendrocyte degeneration—though mechanistic studies with rodents would be needed to confirm such a relationship. Compared to isotropic MRE, anisotropic MRE adds the ability to differentiate between parallel and perpendicular stiffness that will likely offer sensitivity and specificity to how both the axonal and extra-axonal environments contribute to age-related WM changes.

Our findings align with an extensive literature investigating age-related changes in WM microstructure through diffusion MRI, where lower fractional anisotropy and higher mean and radial diffusivity are indicative of microstructural degradation in WM in older adults (Pfefferbaum et al., 2000). Subsequent research consistently revealed regional vulnerabilities, with an anterior-posterior gradient of susceptibility (Davis et al., 2009; Head et al., 2004) and greater differences in WM tracts which connect to the prefrontal cortex (Michielse et al., 2010), consistent with the “last in, first out” hypothesis, wherein anterior tracts that mature latest during development show the earliest age-related decline (Davis et al., 2009; Lebel et al., 2012). While our data suggest lower mechanical properties throughout WM in the older group, especially tensile anisotropy, anterior WM appears to be most strongly or consistently affected, that is, from results of the TBSS analysis of stiffness. We note that the anterior thalamic radiation and forceps minor—both anterior tracts—were the only regions we examined to exhibit significantly lower perpendicular stiffness and parallel shear stiffness, shear anisotropy, and tensile anisotropy in the older group.

One previous study used an anisotropic MRE scheme to study brain stiffness during aging. Kalra et al. (2019) analyzed subjects aged 18–62 years using a 60 Hz MRE acquisition with 2.5 mm voxel resolution. However, they did not find significant correlations with age for anisotropic parameters in any of their WM regions of interest, and instead only showed lower stiffness with age in gray matter but without a difference in anisotropy. Their anisotropic MRE approach is based on direct inversion with a large number of anisotropic parameters, which may induce uncertainty in estimated parameters. Conversely, our TI-NLI approach can effectively describe both the heterogeneity in properties and fiber direction and recovers only a minimal number of parameters, as described in previous studies (Jyoti et al., 2022; McGarry et al., 2022). Additionally, the use of TI-NLI with multi-excitation has been well-characterized and shown to yield repeatable results in WM tracts (D. R. Smith et al., 2022). This robust methodology, and improved resolution, enhances the reliability of our findings and provides a more comprehensive understanding of the differences in anisotropic mechanical properties of WM tracts with age.

Wang et al. (2023) also considered age-related effects on anisotropic MRE parameters, specifically in minipig brains during development. That study employed TI-NLI and an actuator modified from Guertler et al. (2018) to generate multiple wave fields, similar to the human multi-excitation MRE setup (Guertler et al., 2018). They observed higher shear stiffness, shear anisotropy, and tensile anisotropy in white matter compared to gray matter. However, they found only a small, statistically significant increase in substrate shear stiffness with age, and no significant changes in shear or tensile anisotropy. Notably, tensile anisotropy exhibited a slight, non-significant decreasing trend with age, though anisotropy might be expected to increase during development due to rapid myelination at this stage. The authors speculated that this trend might be linked to changes in myelination and cytoskeletal reorganization. Compared to more consistent findings of reduced brain stiffness in aging, the mechanical properties of the developing brain appear complex and are less understood. Previous studies have explored these developmental changes in human brains (McIlvain, Schneider, et al., 2022; McIlvain et al., 2018; Ozkaya et al., 2021), focusing on the mechanical property maturation from adolescence to adulthood. Similarly, Yeung et al. (2019) explored the complexities of brain development and its relationship with mechanical properties, adding to the understanding of how viscoelasticity evolves during early life. These studies highlight how the rapid brain development during childhood and adolescence can affect brain stiffness, though without a clear understanding of how mechanical anisotropy develops in this time.

A common question in the field of brain MRE is whether mechanical property estimates are unique from other microstructural imaging metrics such as those from diffusion MRI, especially in WM where FA, MD, and RD are commonly used as measures of WM structure in healthy aging and disease (Assaf & Pasternak, 2008; Lebel et al., 2012; Raghavan et al., 2021; Sexton et al., 2014). Our data suggest that MRE measures are not wholly redundant with diffusion MRI measures and likely provide unique, complementary information in assessing WM, as evidenced by results from our logistic regression analysis. Even though both MRE and diffusion MRI are sensitive to WM microstructure, and both are affected by aging processes (Davis et al., 2009), we can interpret these findings as likely arising from differences in sensitivity to varying aspects of the underlying microstructure between the methods. Age-related remodeling of white matter likely influences anisotropic mechanical properties through multi-scale microstructural changes. Intra-axonal remodeling, including myelin lamellae loosening and delamination with myelin debris accumulation, plausibly alters lamellar stiffness (Peters, 2002) and thus the stiffness of the axon unit that gives rise to mechanical anisotropy. Aging-related ultrastructural changes observed with electron microscopy, including loss of compact myelin, enlarged inter-axonal gaps, and change in axon size distribution through loss of small axons and preservation of large axons, change neural tissue geometry that likely manifests as changes in tissue mechanics (Atashgar et al., 2025; Saeidi et al., 2023; Wu et al., 2022; Yousefsani et al., 2018). Histology-informed micromechanical models that incorporate both axons and glia in neural tissue microstructure predict that axon density and myelin elasticity are key determinants of anisotropic stiffness, with increased extracellular spacing dampening shear moduli and reducing anisotropy (Chavoshnejad et al., 2021; Saeidi et al., 2023; Wu et al., 2022). Together, these lines of evidence suggest that anisotropic MRE can capture mechanical consequences of age-related microstructural remodeling distinct from diffusion MRI metrics.

These results also provide evidence that mechanical anisotropy estimates from TI-NLI are not strongly influenced by the underlying data from diffusion MRI used during inversion. This finding is consistent with previous work by McGarry et al. (2022) that demonstrated that adding noise to input eigenvector directions did not overly corrupt TI-NLI property estimates. While lower FA values due to aging could potentially introduce variability in the estimated diffusion tensor direction used for inversion, the previous study showed that even with significant noise added to the diffusion signal, the impact on anisotropy estimates remained negligible, with only minor decreases occurring under extreme conditions. Therefore, it is unlikely that the reduced FA values associated with aging would systematically bias MRE anisotropy parameters.

A limitation of the present study is the cross-sectional design and modest sample size (20 younger adults, 18 older adults). Longitudinal, repeated anisotropic MRE measurements across the lifespan in a larger cohort would be necessary to fully characterize the trajectories of WM mechanical anisotropy changes with aging. We compared the anisotropic MRE parameters with multiple established diffusion MRI markers, including FA, MD, RD, and MK, but future studies on WM tracts could examine relationships between MRE and additional diffusion metrics, such as from crossing fiber models or directional kurtosis measures (e.g., axial or radial kurtosis). Our current anisotropic MRE approach with TI-NLI assumes a single fiber direction per voxel, though future implementations may adopt mechanical models that consider multiple fibers (Hou et al., 2021; Wang et al., 2024). Furthermore, how anisotropy and stiffness reflect the underlying mechanisms related to aging, such as demyelination, oligodendrocyte death, or axonal death, remains unclear and animal studies are needed that can study mechanical and cellular metrics simultaneously to understand the sensitivity of MRE outcomes.

## 5 Conclusion

This study demonstrates the capability of anisotropic MRE to characterize age-related differences in the mechanical properties of WM tracts in the human brain. These differences in anisotropic mechanical properties likely reflect underlying microstructural changes associated with aging, such as demyelination, axonal degeneration, and extracellular matrix changes. Importantly, the observed differences in mechanical anisotropy provide complementary information to traditional stiffness metrics alone, suggesting that anisotropic MRE contributes a unique perspective to the estimation of WM related signals, alongside established metrics from diffusion tensor imaging. Ultimately, this multimodal approach may lead to improved diagnostic and prognostic capabilities for age-related neurodegenerative conditions and other neurological disorders affecting WM in the elderly. Future longitudinal studies with larger sample sizes are warranted to further elucidate the trajectories of anisotropic mechanical property changes across the lifespan and their potential clinical implications and demonstrate the additional value of anisotropic MRE in neuroimaging protocols.

## Supplementary Material

Supplementary Material

## Data Availability

Numerical data will be made available by simple request. Raw image files will be made available by request that includes a formal project outline and a data-sharing agreement. MRE sequence code and NLI processing code is available via material transfer agreement.
