# Supplementary material for: Mechanical properties of white matter tracts in aging assessed via anisotropic MR elastography
Source: Imaging Neurosci (Camb). 2026 Mar 5;4:IMAG.a.1156. doi: 10.1162/IMAG.a.1156 (PMC12964139; doi:10.1162/IMAG.a.1156)
Supplement: Supplementary Material [file IMAG.a.1156_supp.pdf]

Mechanical Properties of White Matter Tracts in Aging Assessed via Anisotropic MR Elastography

Diego A. Caban-Rivera, L. Tyler Williams, Matthew D. J. McGarry, Daniel R. Smith, Elijah E. W. Van Houten, Keith D. Paulsen, Philip V. Bayly, Curtis L. Johnson

Supplemental Information

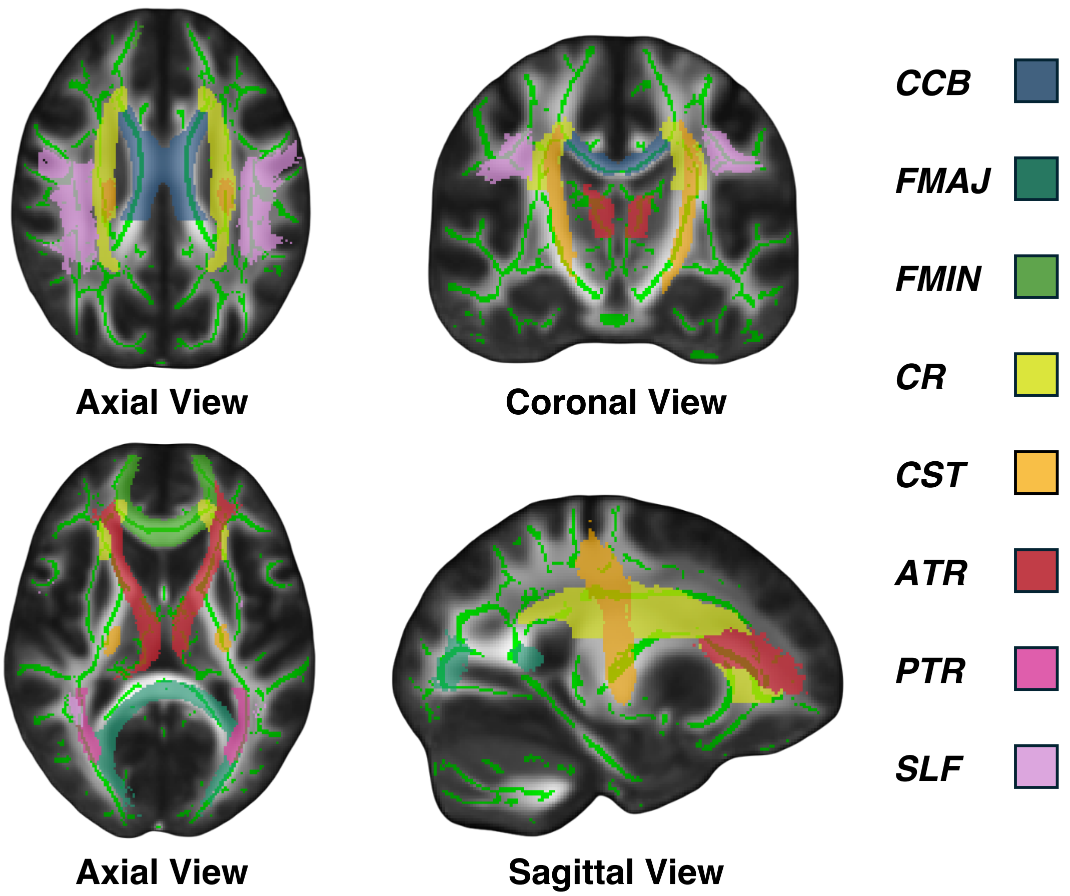

Figure S1: Visualization for different slice orientations of the mean FA map from the FMRIB_FA58 atlas (greyscale), the mean FA skeleton mask (green), and the color-coded white matter tract regions of interest (ROIs) used in the analysis. ROIs were determined by multiplying the white matter skeleton mask with tract masks from probability and tractography atlases resulting in skeletonized ROIs. CCB – Corpus Callosum Body (Denim Blue), FMAJ – Forceps Major (Seafoam Green), FMIN – Forceps Minor (Kelly Green), CR – Corona Radiata (Yellow), CST – Corticospinal Tract (Orange), ATR – Anterior Thalamic Radiation (Red), PTR – Posterior Thalamic Radiation (Magenta), SLF – Superior Longitudinal Fasciculus (Pink).

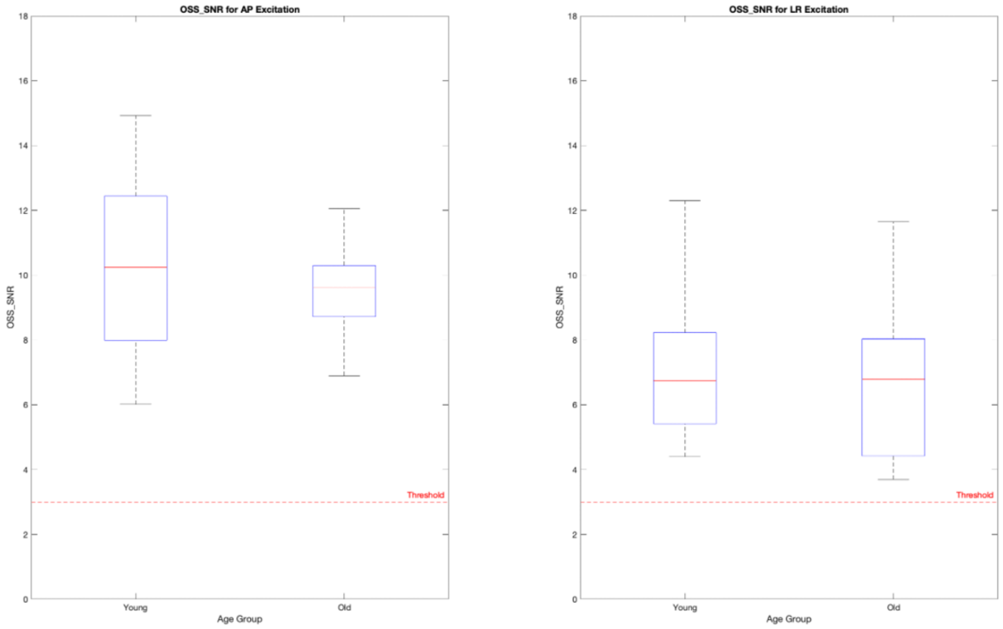

Figure S2: Octahedral shear strain signal-to-noise ratio (OSS-SNR) for anterior-posterior (AP) and left-right (LR) excitations for younger and older adult groups. All datasets from all excitations had OSS-SNR values above the established threshold of 3.0 for stable inversion. There were no significant differences in OSS-SNR between YA and OA groups for either AP (YA: 10.78 ± 3.39, OA: 9.55 ± 1.45; $p = 0.171$) or LR (YA: 7.21 ± 2.39, OA: 6.83 ± 2.50; $p = 0.633$) excitation.

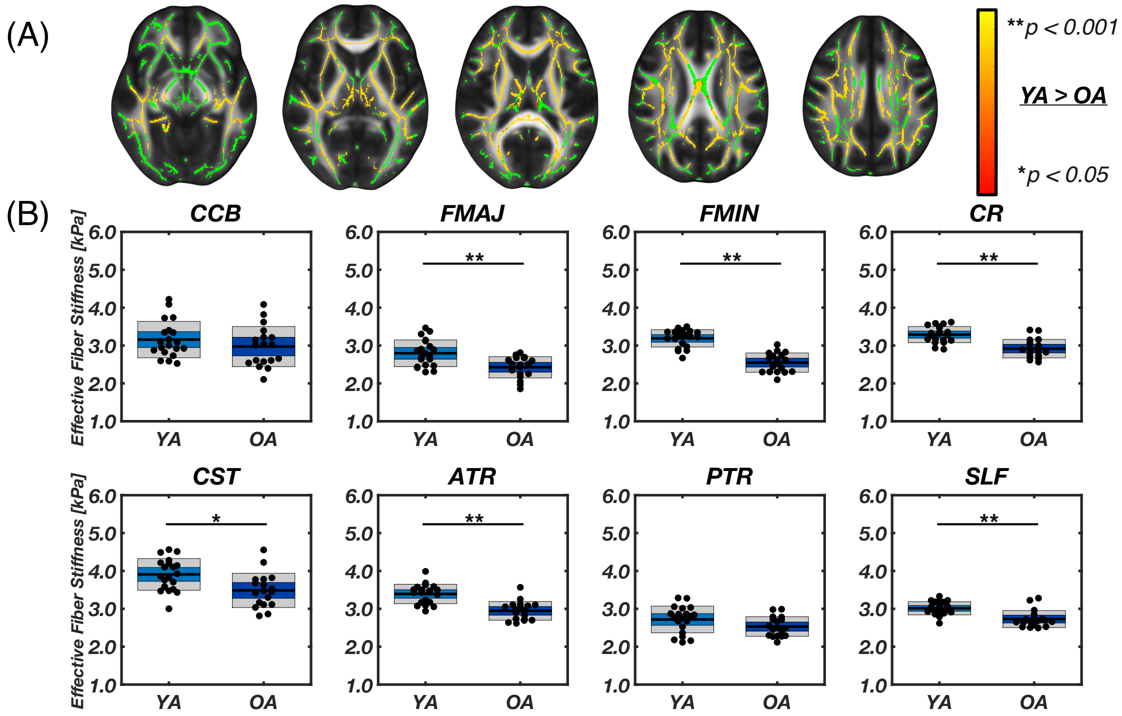

Figure S3: A) Results from voxel-wise analysis using tract based spatial statistics (TBSS) for axial slices [66, 77, 86, 98, 107] including the white matter skeleton mask in green and the contrast of Young Adult > Older Adult for shear stiffness parallel to the fibers (μ_1) overlaid by the red-yellow color bar, which represents voxel-wise p-values between 0.05-0.001. The OA > YA analysis contrast did not result in significant voxels. B) Segmented white matter region averages of μ_1. Asterisks (*) indicate significant differences between groups as determined by two-sample Student's t-tests with Bonferroni correction (* $p < 0.00625$, ** $p < 0.001$). Older adults had significantly lower μ_1 in most tracts: forceps major (FMAJ, p<0.001**), forceps minor (FMIN, p<0.001**), corona radiata (CR, p<0.001**), corticospinal tract (CST, p=0.0054*), anterior thalamic radiation (ATR, p<0.001**), and superior longitudinal fasciculus (SLF, p<0.001**).

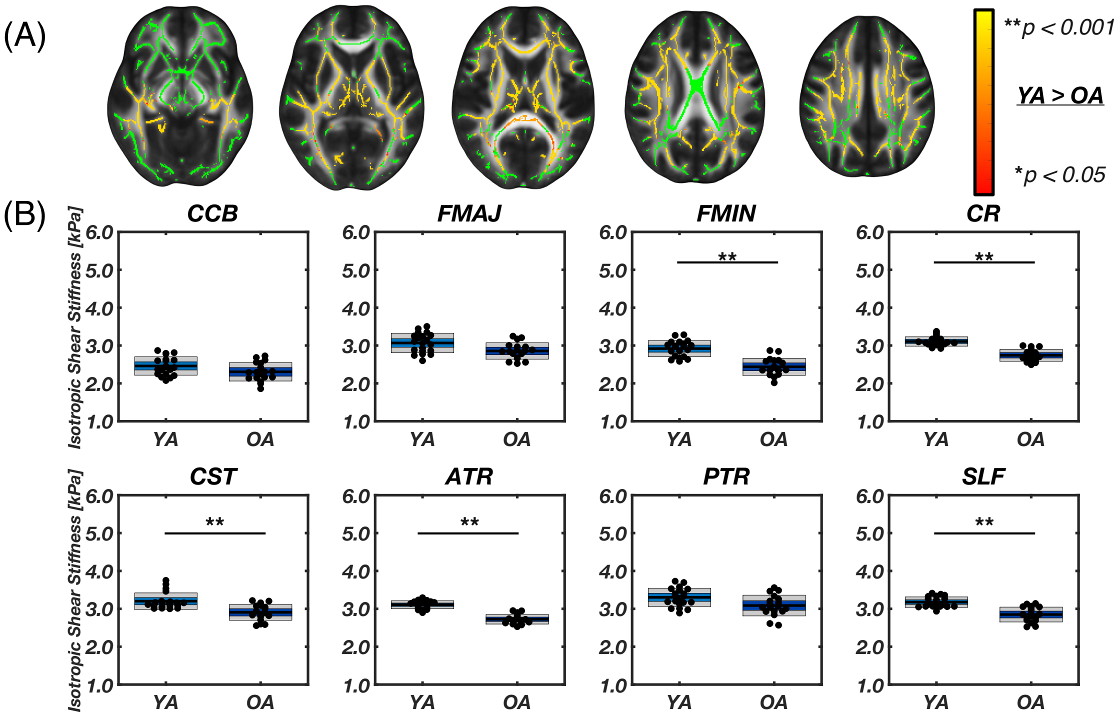

Figure S4: A) Results from voxel-wise analysis using tract based spatial statistics (TBSS) for axial slices [66, 77, 86, 98, 107] including the white matter skeleton mask in green and the contrast of Young Adult > Older Adult for isotropic shear stiffness (μ_{iso}) overlaid by the red-yellow color bar, which represents voxel-wise p-values between 0.05-0.001. The OA > YA analysis contrast did not result in significant voxels. B) Segmented white matter region averages of μ_{iso}. Asterisks (*) indicate significant differences between groups as determined by two-sample Student's t-tests with Bonferroni correction (* p < 0.00625, ** p < 0.001). Older adults had significantly lower μ_{iso} in multiple tracts: forceps minor (FMIN, p<0.001**), corona radiata (CR, p<0.001**), corticospinal tract (CST, p<0.001**), anterior thalamic radiation (ATR, p<0.001**), and superior longitudinal fasciculus (SLF, p<0.001**).

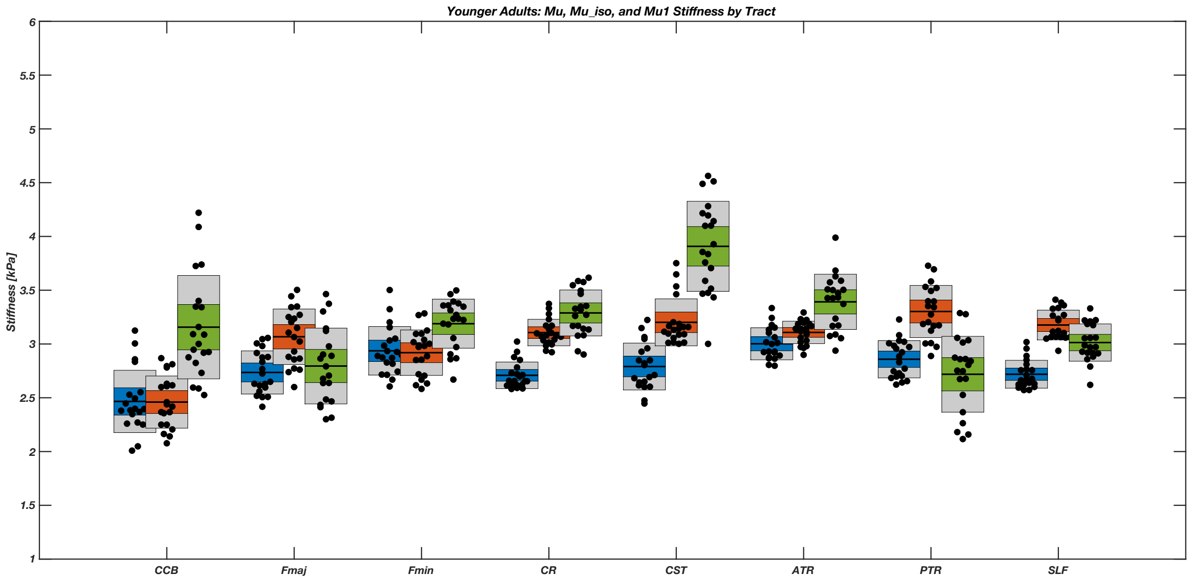

Figure S5: Segmented white matter ROI averages of substrate shear stiffness, isotropic shear stiffness, and shear stiffness parallel to the fibers, for all younger adults (n = 20, mean age: 25). For most tracts, the substrate shear stiffness (μ_2, Blue) was the lowest, with either isotropic (μ_{iso}) or parallel stiffness (μ_1) being highest in each of the tracts.

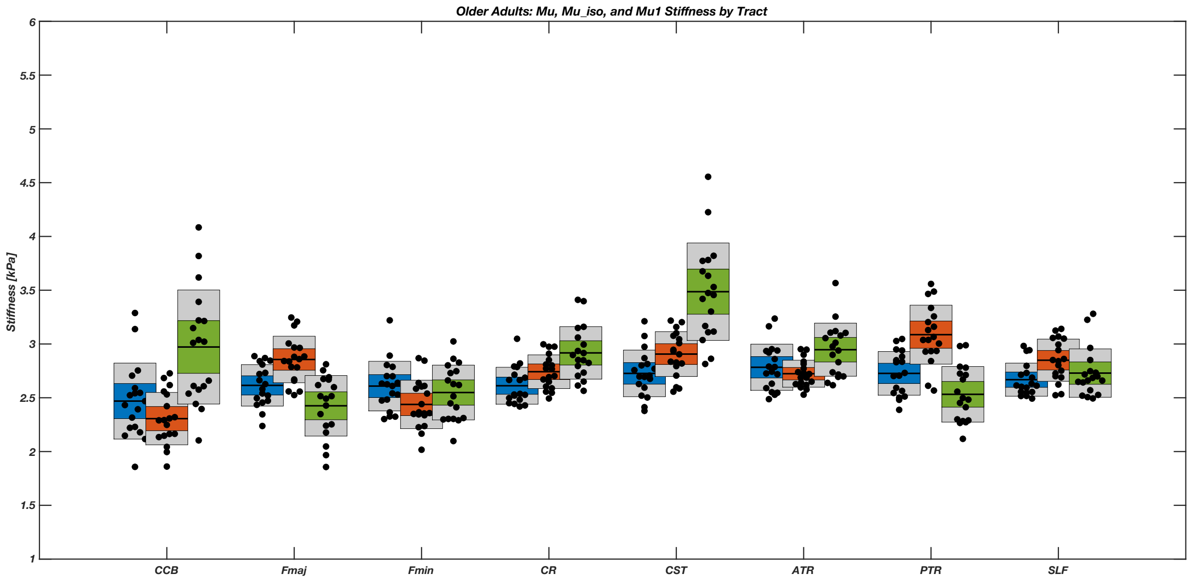

Figure S6: Segmented white matter ROI averages of substrate shear stiffness, isotropic shear stiffness, and shear stiffness parallel to the fibers, for all older adults (n = 18, mean age: 68). For most tracts, the substrate shear stiffness (μ_2) was the lowest, with isotropic (μ_{iso}) or parallel stiffness (μ_1) being highest in each of the tracts.

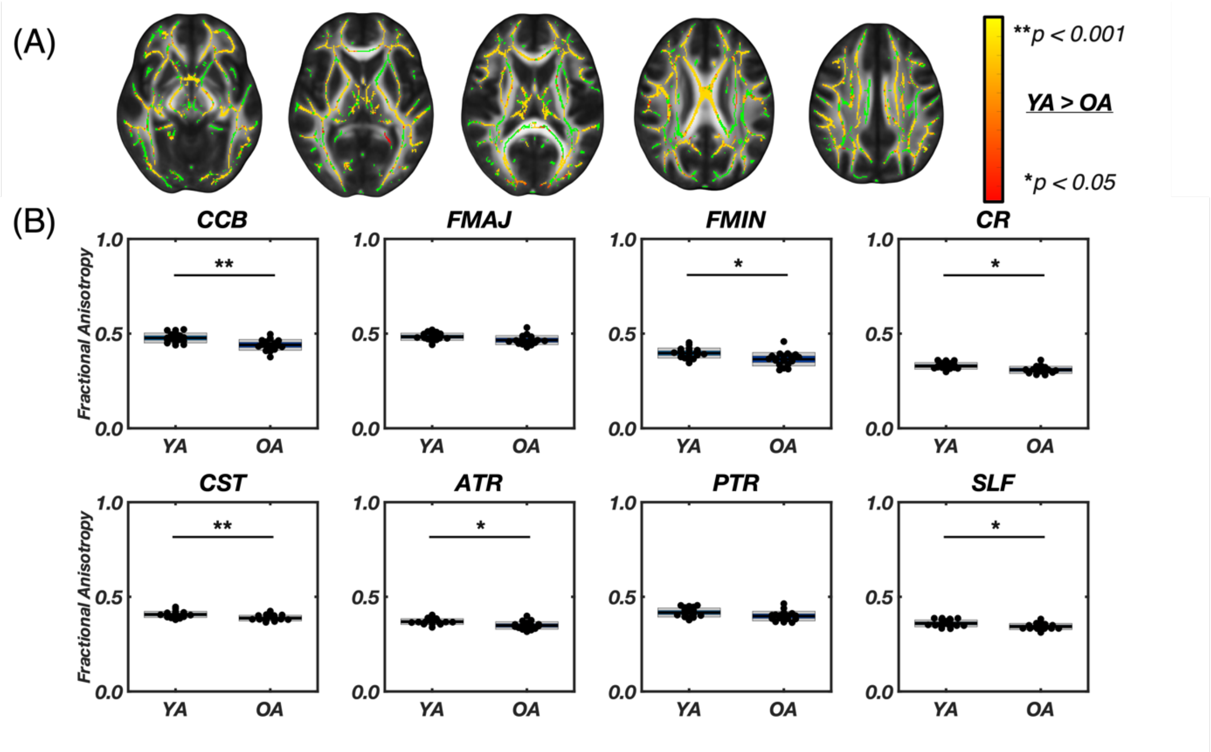

Figure S7: A) Results from voxel-wise analysis using tract based spatial statistics (TBSS) for axial slices [66, 77, 86, 98, 107] including the white matter skeleton mask in green and the contrast of Young Adult > Older Adult for fractional anisotropy (FA) overlaid by the red-yellow color bar, representing voxel-wise p-values between 0.05-0.001. The OA > YA analysis contrast did not result in significant voxels. B) Segmented white matter region averages of FA. Asterisks (*) indicate significant differences between groups as determined by two-sample Student's t-tests with Bonferroni correction (* $p < 0.00625$, ** $p < 0.001$). Older adults had significantly lower FA in the corpus callosum body (CCB, p<0.001**), forceps minor (FMIN, p=0.0040*), corona radiata (CR, p=0.0018*), corticospinal tract (CST, p<0.001**), anterior thalamic radiation (ATR, p=0.0017*), and superior longitudinal fasciculus (SLF, p=0.0057*).

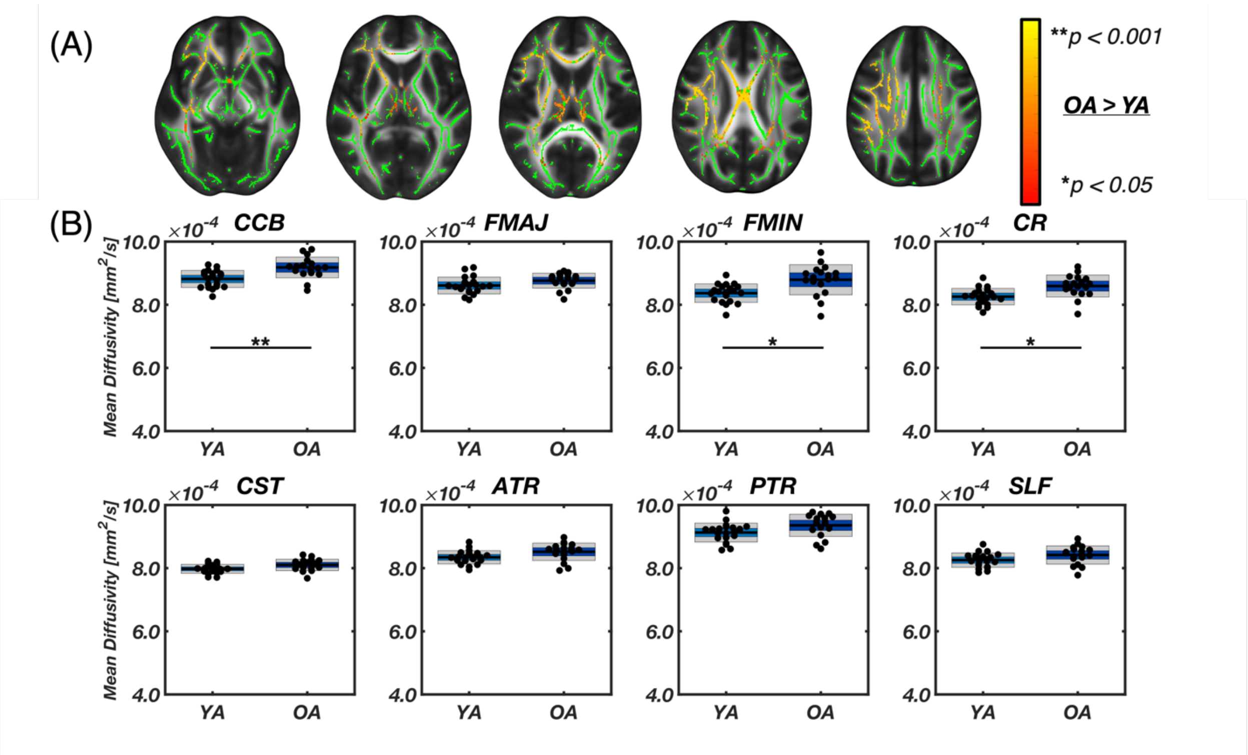

Figure S8: A) Results from voxel-wise analysis using tract based spatial statistics (TBSS) for axial slices [66, 77, 86, 98, 107] including the white matter skeleton mask in green and the contrast of Older Adult > Younger Adult for mean diffusivity (MD) overlaid by the red-yellow color bar, representing voxel-wise p-values between 0.05-0.001. The YA > OA analysis contrast did not result in significant voxels. B) Segmented white matter region averages of MD. Asterisks (*) indicate significant differences between groups as determined by two-sample Student's t-tests with Bonferroni correction (* $p < 0.00625$, ** $p < 0.001$). Older adults had significantly higher MD in the corpus callosum body (CCB, $p<0.001$**), forceps minor (FMIN, $p=0.0029$*), and corona radiata (CR, $p=0.0022$*).

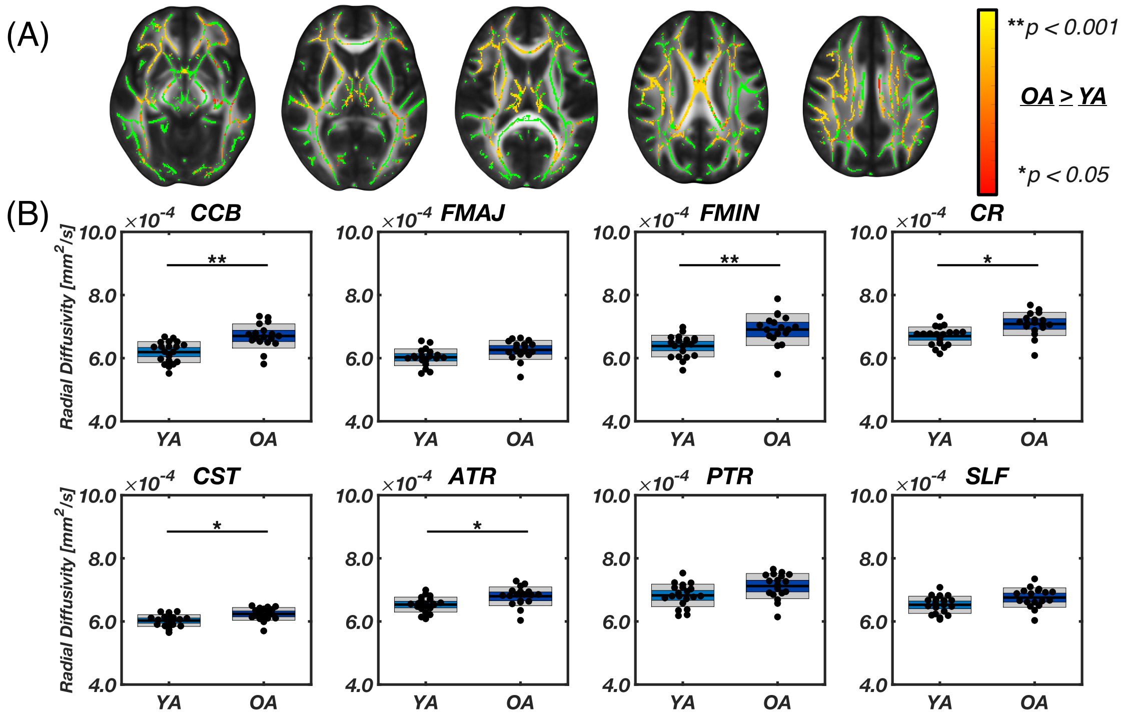

Figure S9: A) Results from voxel-wise analysis using tract based spatial statistics (TBSS) for axial slices [66, 77, 86, 98, 107] including the white matter skeleton mask in green and the contrast of Older Adult > Younger Adult for radial diffusivity (RD) overlaid with the red-yellow color bar, which represents voxel-wise p-values between 0.05-0.001. No significant voxels were found in the YA > OA contrast. B) Segmented white matter region averages of RD. Asterisks (*) indicate significant differences between groups as determined by two-sample Student's t-tests with Bonferroni correction (* $p < 0.00625$, ** $p < 0.001$). Older adults had significantly higher RD in the corpus callosum body (CCB, $p<0.001$**), forceps minor (FMIN, $p<0.001$**), corona radiata (CR, $p=0.0012$*), corticospinal tract (CST, $p=0.0021$*), and anterior thalamic radiation (ATR, $p=0.0052$*).

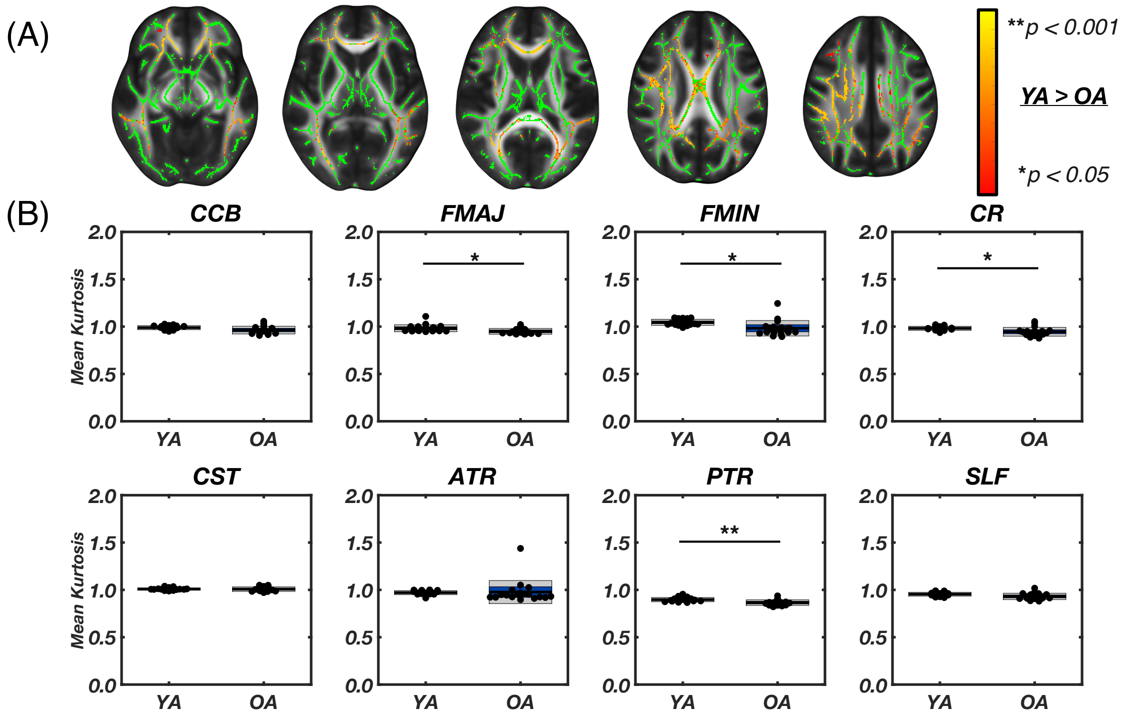

Figure S10: A) Results from voxel-wise analysis using tract based spatial statistics (TBSS) for axial slices [66, 77, 86, 98, 107], showing the white matter skeleton mask in green and the contrast of Young Adult > Older Adult for mean kurtosis (MK) overlaid with the red-yellow color bar, which represents voxel-wise p-values between 0.05-0.001. No significant voxels were found in the OA > YA contrast. B) Segmented white matter region averages of MK. Asterisks (*) indicate significant differences between groups as determined by two-sample Student's t-tests with Bonferroni correction (* $p < 0.00625$, ** $p < 0.001$). Older adults had significantly lower MK in the forceps major (FMAJ, p=0.0062*), forceps minor (FMIN, p=0.0061*), corona radiata (CR, p=0.0062*), and posterior thalamic radiation (PTR, p<0.001**).

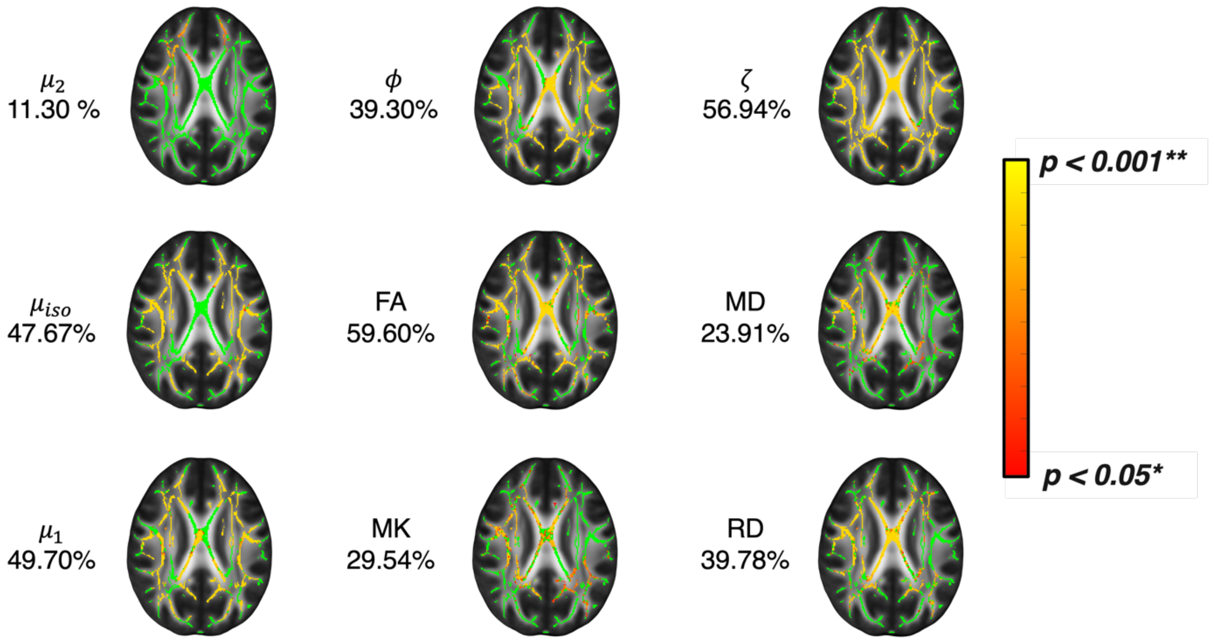

Figure S11: Summary of TBSS voxel-wise statistical analysis for mechanical parameters (μ_2, μ_1, μ_{iso}, ϕ, ζ) and DTI parameters (FA, MD, RD, MK) of the younger adult versus older adult contrasts. Substrate shear stiffness had the smallest percentage of significant voxels within the cerebrum white matter. Stiffness parallel to the fibers and isotropic stiffness both had larger percentages, more comparable to FA and RD. Shear and tensile anisotropy had larger percentages than MD and MK but comparable with FA and RD.

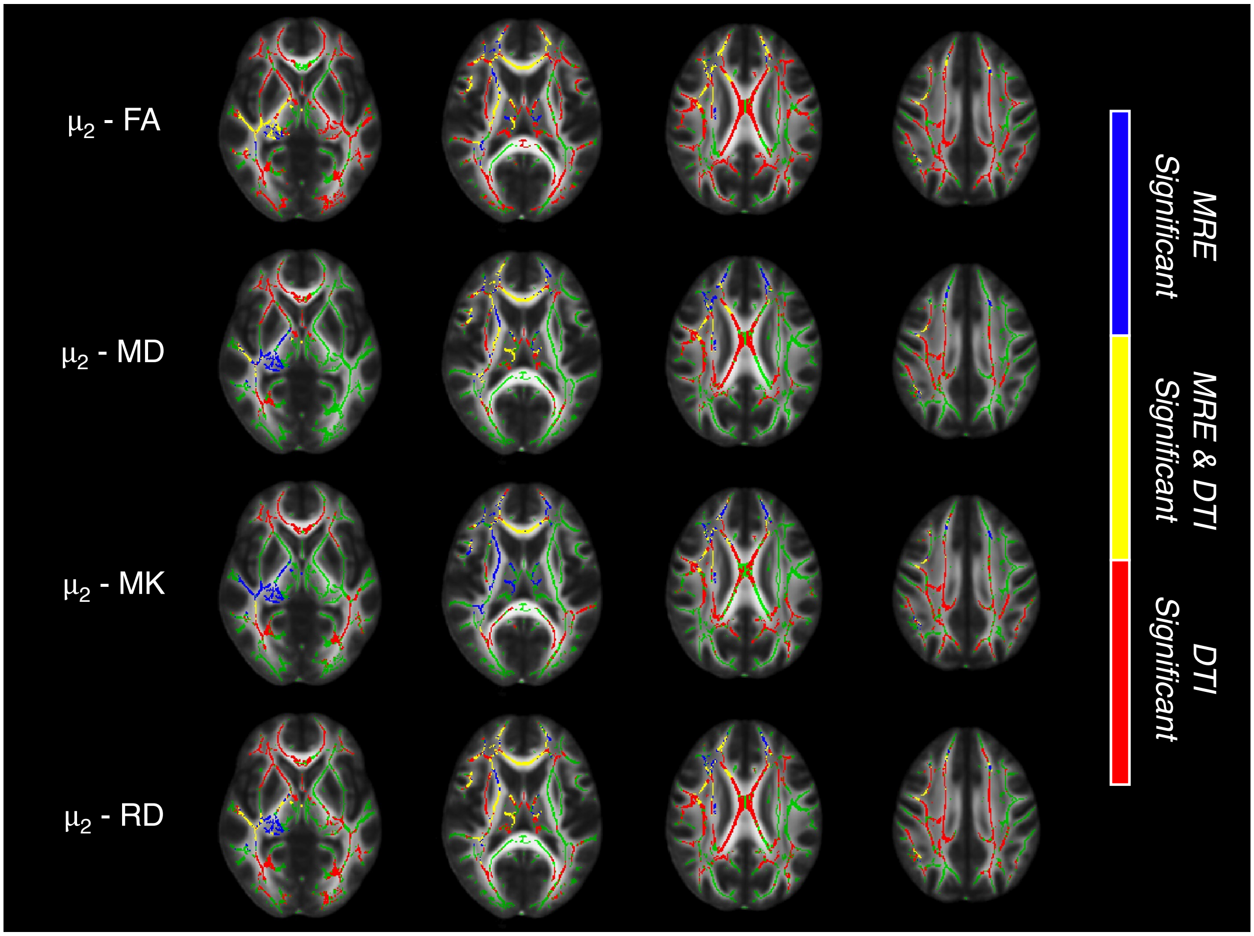

Figure S12: Comparison of areas with significant voxels resulting from contrasting younger versus older adults, between MRE parameter μ_2 and DTI parameters FA, MD, MK, and RD. The background image is the mean fractional anisotropy atlas overlaid by the white matter skeleton mask in green. Three colors are shown to distinguish 1) voxels in which μ_2 is significant only, 2) voxels in which DTI parameters are significant only, 3) both μ_2 and DTI parameters are significant (where they overlap). Areas where neither MRE nor DTI parameters are significant do not have a color overlaid over the mask (show up green).

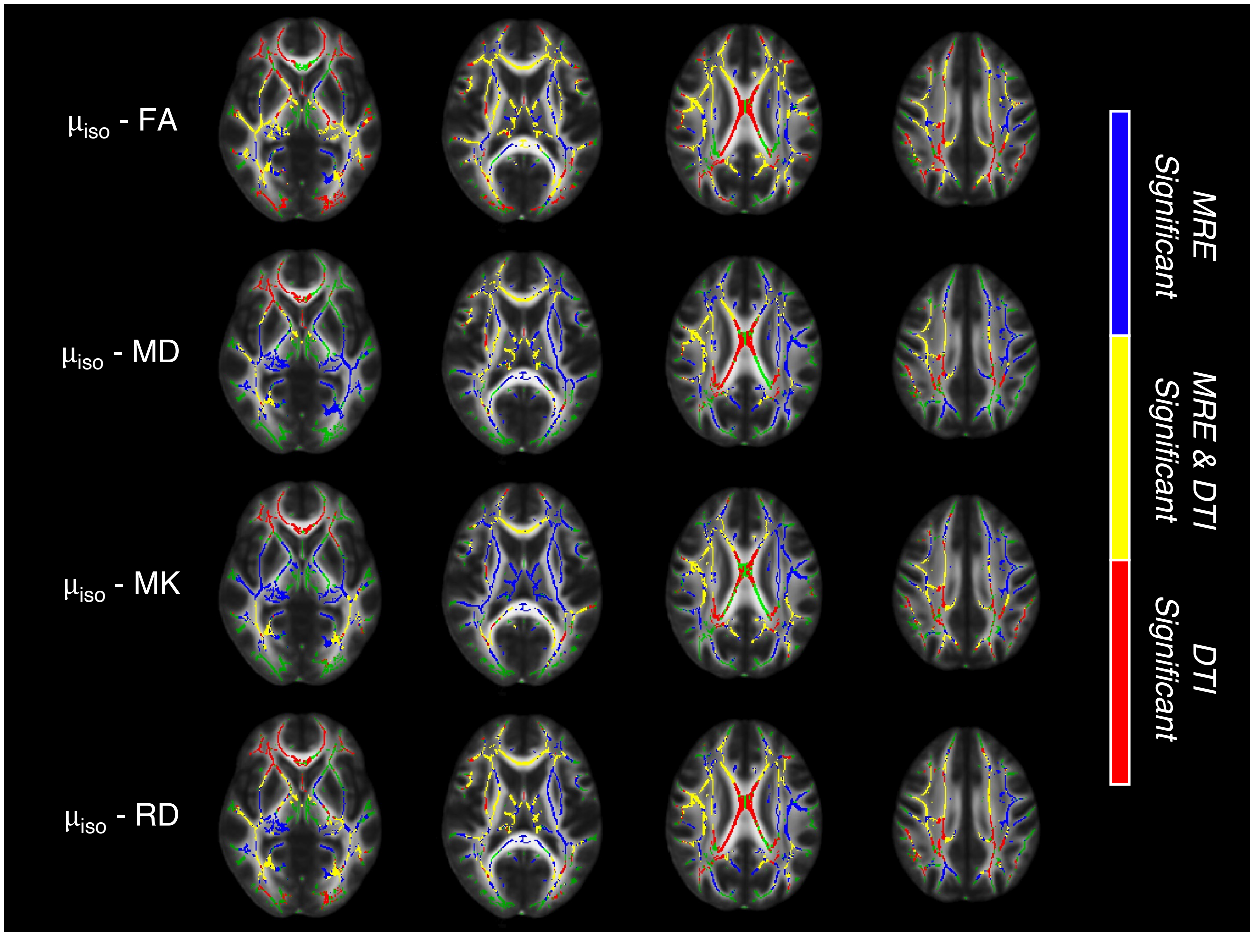

Figure S13: Comparison of areas with significant voxels resulting from contrasting younger versus older adults, between MRE parameter μ_{iso} and DTI parameters FA, MD, MK, and RD. The background image is the mean fractional anisotropy atlas overlaid by the white matter skeleton mask in green. Three colors are shown to distinguish 1) voxels in which μ_{iso} is significant only, 2) voxels in which DTI parameters are significant only, 3) both μ_{iso} and DTI parameters are significant (where they overlap). Areas where neither MRE nor DTI parameters are significant do not have a color overlaid over the mask (show up green).

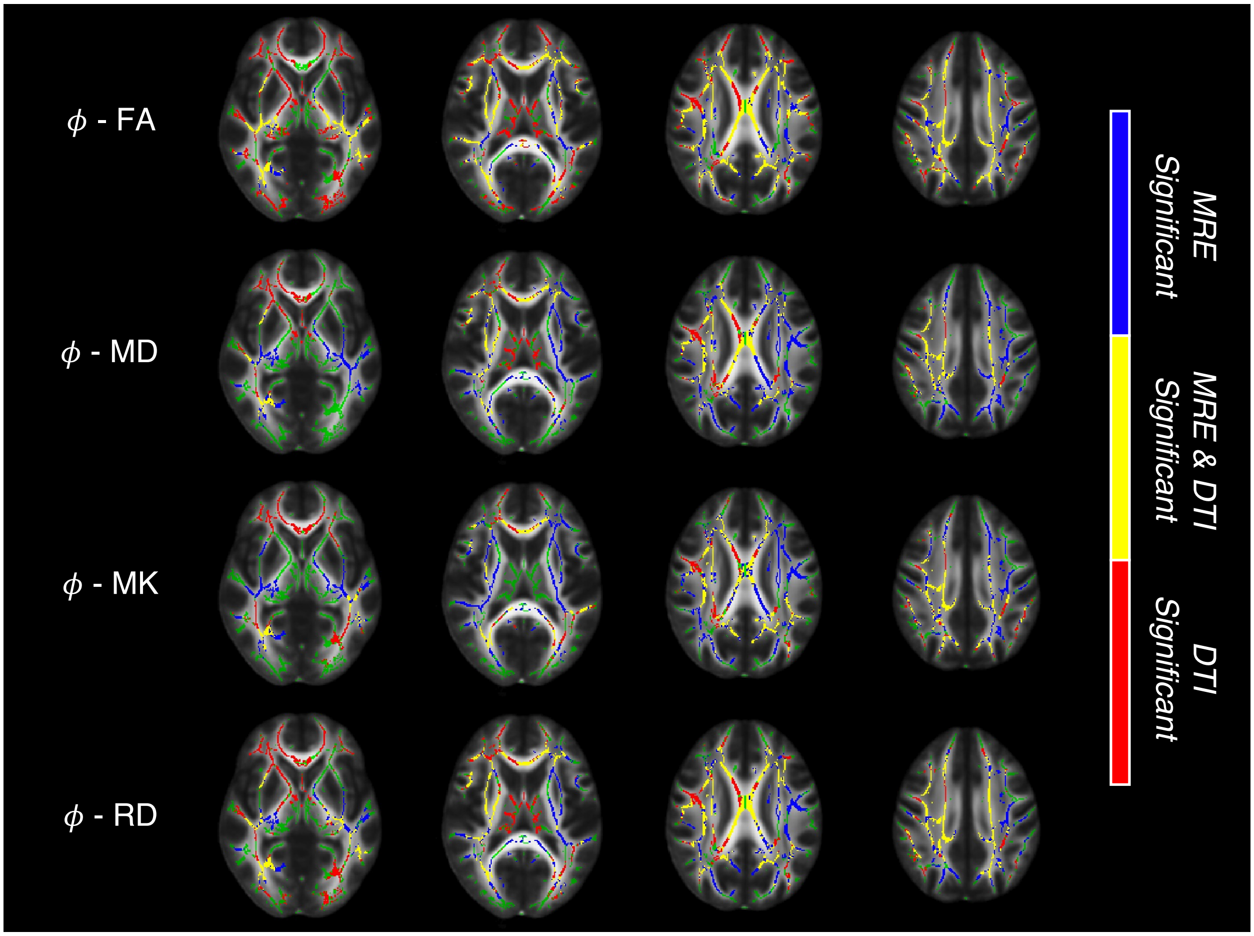

Figure S14: Comparison of areas with significant voxels resulting from contrasting younger versus older adults, between MRE parameter ϕ (shear anisotropy) and DTI parameters FA, MD, MK, and RD. The background image is the mean fractional anisotropy atlas overlaid by the white matter skeleton mask in green. Three colors are shown to distinguish 1) voxels in which ϕ is significant only, 2) voxels in which DTI parameters are significant only, 3) both ϕ and DTI parameters are significant (where they overlap). Areas where neither MRE nor DTI parameters are significant do not have a color overlaid over the mask (show up green).

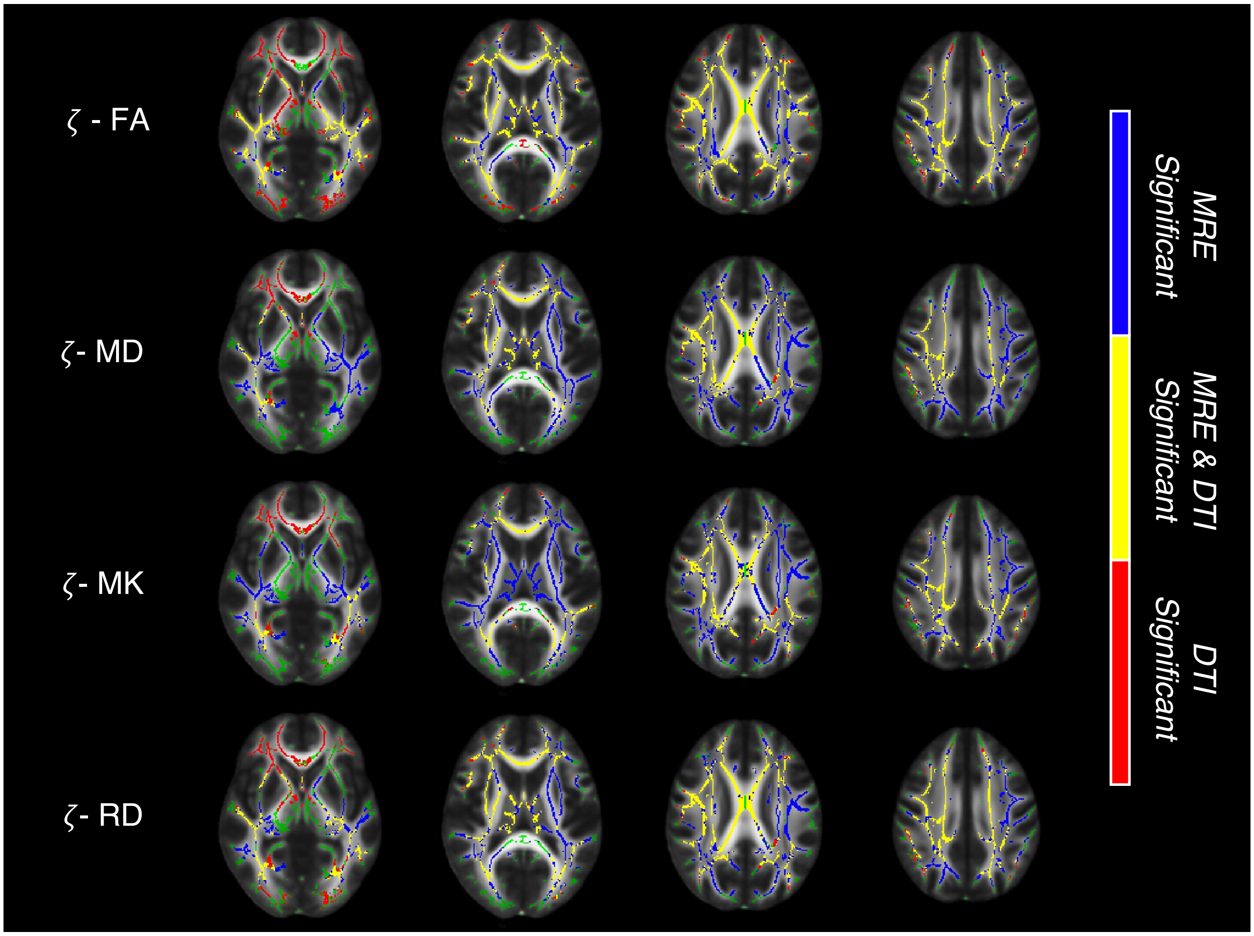

Figure S15: Supplementary Figure X: Comparison of areas with significant voxels resulting from contrasting younger versus older adults, between MRE parameter ζ (shear anisotropy) and DTI parameters FA, MD, MK, and RD. The background image is the mean fractional anisotropy atlas overlaid by the white matter skeleton mask in green. Three colors are shown to distinguish 1) voxels in which ζ is significant only, 2) voxels in which DTI parameters are significant only, 3) both ζ and DTI parameters are significant (where they overlap). Areas where neither MRE nor DTI parameters are significant do not have a color overlaid over the mask (shown in green).

Supplementary Table 1: Overview of μ_1 and μ_{iso} property means and standard deviations in white matter tracts for young adults (YA) versus older adults (OA). Statistically significant group differences after Bonferroni correction are bolded and starred for significance level **p < 0.00625**.

Region	μ_1, YA [kPa]	μ_1, OA [kPa]	P-values	μ_{iso}, YA [kPa]	μ_{iso}, OA [kPa]	P-values
**CCB**	3.16±0.48 (2.53-4.22)	2.97±0.53 (2.10-4.08)	p = 0.271	2.46±0.24 (2.08-2.87)	2.31±0.24 (1.86-2.73)	p = 0.059
**FMAJ**	2.80±0.35 (2.30-3.46)	2.43±0.28 (1.86-2.81)	**p < 0.001****	3.07±0.26 (2.60-3.50)	2.86±0.22 (2.53-3.25)	p = 0.009
**FMIN**	3.19±0.23 (2.67-3.50)	2.55±0.25 (2.10-3.02)	**p < 0.001****	2.92±0.21 (2.58-3.28)	2.44±0.23 (2.02-2.87)	**p < 0.001****
**CR**	3.29±0.21 (2.90-3.62)	2.92±0.24 (2.56-3.41)	**p < 0.001****	3.11±0.12 (2.92-3.37)	2.74±0.16 (2.49-3.00)	**p < 0.001****
**CST**	3.91±0.42 (3.00-4.56)	3.49±0.45 (2.81-4.55)	**p = 0.005***	3.20±0.22 (3.00-3.75)	2.91±0.21 (2.56-3.22)	**p < 0.001****
**ATR**	3.39±0.26 (2.94-3.99)	2.95±0.25 (2.62-3.57)	**p = 0.001***	3.11±0.10 (2.90-3.29)	2.72±0.13 (2.53-2.95)	**p < 0.001****
**PTR**	2.72±0.35 (2.12-3.29)	2.53±0.26 (2.12-2.99)	p = 0.070	3.30±0.24 (2.89-3.73)	3.09±0.27 (2.57-3.56)	p = 0.015
**SLF**	3.01±0.17 (2.62-3.33)	2.73±0.23 (2.49-3.28)	**p < 0.001****	3.18±0.14 (2.94-3.41)	2.85±0.20 (2.52-3.14)	**p < 0.001****

Supplementary Table 2: Overview of DTI parameter means and standard deviations in white matter tracts for young adults (YA) versus older adults (OA).

Region	FA, YA	FA, OA	RD, YA [μm²/s]	RD, OA [μm²/s]	MK, YA	MK, OA	MD, YA [μm²/s]	MD, OA [μm²/s]
**CCB**	0.48±0.03 (0.44-0.52)	0.44±0.03 (0.38-0.50)	0.619±0.033 (0.552-0.668)	0.670±0.038 (0.581-0.733)	0.99±0.02 (0.95-1.03)	0.96±0.04 (0.91-1.06)	0.882±0.027 (0.826-0.927)	0.918±0.033 (0.845-0.976)
**FMAJ**	0.48±0.02 (0.44-0.52)	0.47±0.02 (0.43-0.53)	0.603±0.027 (0.552-0.655)	0.626±0.030 (0.540-0.664)	0.98±0.04 (0.95-1.11)	0.95±0.03 (0.92-1.02)	0.861±0.027 (0.815-0.918)	0.876±0.024 (0.817-0.907)
**FMIN**	0.40±0.03 (0.35-0.45)	0.36±0.04 (0.31-0.46)	0.638±0.034 (0.562-0.698)	0.691±0.051 (0.549-0.788)	1.04±0.03 (0.99-1.09)	0.98±0.08 (0.89-1.24)	0.837±0.029 (0.767-0.894)	0.879±0.048 (0.764-0.966)
**CR**	0.33±0.02 (0.30-0.36)	0.31±0.02 (0.28-0.36)	0.670±0.029 (0.614-0.731)	0.708±0.037 (0.608-0.768)	0.98±0.02 (0.94-1.02)	0.95±0.05 (0.88-1.05)	0.826±0.026 (0.776-0.886)	0.859±0.035 (0.771-0.921)
**CST**	0.41±0.02 (0.38-0.45)	0.39±0.02 (0.37-0.42)	0.603±0.019 (0.565-0.632)	0.624±0.020 (0.570-0.650)	1.01±0.01 (0.99-1.04)	1.01±0.02 (0.97-1.05)	0.798±0.014 (0.771-0.822)	0.810±0.018 (0.768-0.842)
**ATR**	0.37±0.02 (0.34-0.40)	0.35±0.02 (0.32-0.40)	0.653±0.024 (0.608-0.700)	0.680±0.030 (0.603-0.728)	0.97±0.02 (0.91-1.00)	0.98±0.12 (0.90-1.44)	0.834±0.021 (0.794-0.883)	0.852±0.028 (0.793-0.897)
**PTR**	0.42±0.02 (0.38-0.45)	0.40±0.03 (0.36-0.46)	0.683±0.036 (0.618-0.753)	0.712±0.040 (0.614-0.766)	0.90±0.02 (0.87-0.95)	0.87±0.03 (0.82-0.94)	0.913±0.030 (0.857-0.981)	0.936±0.035 (0.861-0.978)
**SLF**	0.36±0.02 (0.33-0.39)	0.34±0.02 (0.31-0.38)	0.653±0.027 (0.607-0.708)	0.676±0.031 (0.603-0.735)	0.95±0.02 (0.92-0.99)	0.93±0.03 (0.88-1.02)	0.825±0.023 (0.786-0.876)	0.842±0.029 (0.778-0.893)

Supplementary Table 3: Logistic regression results summarized by p-values and β values, for each of the MRE parameters separated by columns, which were added to the regression after mean diffusivity (MD) to classify younger versus older adults. The rows of the table depict the different white matter tract ROIs investigated in the study.

Region	μ_2, p-value	μ_2, β	ϕ, p-value	ϕ, β	ζ, p-value	ζ, β	μ_{iso}, p-value	μ_{iso}, β	μ_1, p-value	μ_1, β
CCB	0.681	0	0.056	9.273	0.014	20.597	0.151	0.002	0.561	0
Fmaj	0.181	0.003	0.024	9.885	0.05	5.698	0.033	0.003	0.01	0.004
Fmin	0.009	0.007	0.008	36.567	0.008	11.659	0.007	0.009	0.011	0.01
CR	0.092	0.005	0.004	78.439	0.004	17.509	0.017	0.027	0.005	0.007
CST	0.276	0.002	0.002	24.493	0.002	15.152	0.016	0.01	0.022	0.002
ATR	0.012	0.006	0.003	83.811	0.003	14.215	0.108	0.054	0.003	0.007
PTR	0.027	0.005	0.12	7.237	0.023	7.824	0.024	0.003	0.028	0.003
SLF	0.3	0.003	0.002	55.036	0.001	14.759	0.01	0.014	0.002	0.007

Supplementary Table 4: Logistic regression results summarized by p-values and β values, for each of the MRE parameters separated by columns, which were added to the regression after mean kurtosis (MK) to classify younger versus older adults. The rows of the table depict the different white matter tract ROIs investigated in the study.

Region	μ_2, p-value	μ_2, β	ϕ, p-value	ϕ, β	ζ, p-value	ζ, β	μ_{iso}, p-value	μ_{iso}, β	μ_1, p-value	μ_1, β
CCB	0.629	-0.001	0.047	8.553	0.002	14.848	0.1	0.003	0.49	0.001
Fmaj	0.378	0.002	0.052	8.094	0.063	5.438	0.066	0.003	0.02	0.004
Fmin	0.009	0.007	0.004	34.043	0.016	18.638	0.009	0.013	0.009	0.009
CR	0.105	0.005	0.003	80.21	0.004	18.274	0.076	0.038	0.004	0.006
CST	0.355	0.001	0.001	25.048	0.002	15.993	0.007	0.011	0.012	0.002
ATR	0.005	0.007	0.003	58.016	0.002	13.863	0.085	0.038	0.001	0.007
PTR	0.041	0.005	0.384	4.13	0.1	5.587	0.084	0.003	0.11	0.002
SLF	0.292	0.003	0.003	52.418	0.002	14.54	0.009	0.013	0.004	0.007
